# Abnormal Stress Reduced miR‐330 Supplementation Alleviates Osteoarthritis Progression by Suppressing Osteochondral Catabolism

**DOI:** 10.1111/acel.70553

**Published:** 2026-05-29

**Authors:** Luxiang Zou, Kaiwen Yang, Chuyao Wang, Yeke Yu, Xiaoyu Zhang, Chuan Lu, Jieyun Zhao, An Qin, Dongmei He

**Affiliations:** ^1^ Department of Oral Surgery Ninth People's Hospital, Shanghai Jiao Tong University School of Medicine Shanghai China; ^2^ Shanghai Key Laboratory of Stomatology & Shanghai Research Institute of Stomatology National Clinical Research Center of Stomatology Shanghai China; ^3^ Shanghai Key Laboratory of Orthopedic Implants, Department of Orthopedics, Ninth People's Hospital Shanghai Jiao Tong University School of Medicine Shanghai China

**Keywords:** cartilage‐subchondral bone integrated regulation, mechanical overloading, miR‐330, osteoarthritis

## Abstract

Abnormal mechanical stress plays a crucial role in the progression of osteoarthritis (OA). Key factors regulating mechanical response remain to be explored. This study harvested synovium fluids from 96 temporomandibular disorders (TMD) patients and revealed that miR‐330 was significantly reduced under abnormal mechanical stress. Similarly, the expression of miR‐330 is also decreased in cartilage and subchondral bone tissue from OA clinical specimens and animal models. Global knockout of miR‐330 aggravated osteoarthritis development in both temporomandibular joint and knee joint. Conditional knockout of miR‐330 in either chondrocytes or osteoclasts exhibited a similar phenotype in both knee and TMJ mechanical instable animal models, suggesting the importance of miR‐330 in both types of cells. Further molecular mechanism exploration unveiled that miR‐330 can regulate the catabolism of chondrocytes and osteoclasts via suppressing CTGF, FGFR1, and EPOR as well as key inflammatory cytokines such as IL‐1β and TNF‐α. More importantly, intra‐articular supplement of miR‐330 exerts a therapeutic effect by mitigating the detrimental impacts of abnormal mechanical stress, where inhibition of chondrocytes apoptosis, osteoclasts activation, and inflammation were further confirmed via single‐cell RNA sequencing analysis. In conclusion, this study for the first time revealed that miR‐330 is a mechanical responsive, osteoarthritis protective micro RNA. We provide evidence that miR‐330 has potential diagnostic and therapeutic value for both temporomandibular and knee OA.

## Graphical abstract

Downregulation of miR‐330 under abnormal stress exacerbates catabolism in cartilage and subchondral bone, thereby promoting the progression of osteoarthritis.

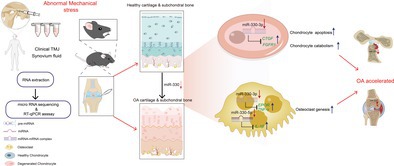

## 1 Introduction

Osteoarthritis (OA) is a prevalent joint disorder characterized by chronic pain and functional impairments, which imposes significant economic burdens on both society and individuals (Ferket et al. 2017; Hunter and Bierma‐Zeinstra 2019). World Health Organization (WHO) has classified osteoarthritis, alongside cardiovascular disease and cancer, among the three most impactful diseases with substantial health implications (Cross et al. 2014). Currently, non‐surgical treatment of OA as nonsteroidal anti‐inflammatory drugs (NSAIDs) and lubricating agents such as glucosamine are insufficient in inhibiting or reversing the progression of OA and lack of specificity (Whittaker et al. 2021). For the advanced OA, surgical treatment and artificial prosthesis replacement are needed. Reversal of the OA progression especially in early stages depends on the elucidation of its pathological and molecular mechanisms.

OA progression is characterized by a complex interplay of pathological degeneration within the articular cartilage and the underlying subchondral bone (Yao et al. 2023; Batarfi et al. 2025). Cartilage breakdown initiates with the disruption of the extracellular matrix, which is driven by chondrocyte hypertrophy and the excessive production of catabolic enzymes, leading to the loss of essential components, diminished hydration, and compromised biomechanical function (Li et al. 2021). Concomitantly, the subchondral bone undergoes significant pathological remodeling, such as initial bone resorption followed by aberrant bone formation, resulting in resorption and sclerosis. Crucially, osteoclasts, the specialized bone‐resorbing cells, play a pivotal yet dysregulated role in this subchondral pathology. Activated osteoclasts are recruited and hyperactivated at the osteochondral junction, largely stimulated by pro‐inflammatory cytokines such as TNF‐α and IL‐1β expressed by macrophages and other cells within the OA joint microenvironment. This heightened osteoclastic activity also contributes significantly to excessive subchondral bone resorption, trabecular thinning, and the release of catabolic factors and matrix‐degrading enzymes that further damage the overlying cartilage (Hu et al. 2021).

Furthermore, abnormal mechanical loading can also synergistically contribute to OA development with increased catabolism activation in cartilage as well as subchondral bone, which has been recognized as one of the most important risk factors (Jiang et al. 2023). Although the complete molecular network underlying its effects remains incompletely understood, research indicates that abnormal mechanical stress activates multiple signaling pathways during OA progression. Mechanical sensitive proteins such as PIEZO1 and TRPV4 played a significant role in cartilage degeneration and dysregulated bone formation in OA as well (Agarwal et al. 2021; Wang et al. 2022). BRD4 inhibition can ameliorate aberrant mechanical stress‐induced temporomandibular joint osteoarthritis (TMJOA) through modulating MLKL and necroptosis as reported (Huang, Han, et al. 2025; Huang, Zheng, et al. 2025). Our previous study also showed that abnormal mechanical stress contributes to the development of TMJOA and changes the protein profiles (Zou et al. 2024). Despite these insights, the specific mechanisms by which mechanical overloading induces the complex, multi‐tissue degeneration require further elucidation.

Apart from the mentioned functional proteins, noncoding RNAs exert versatile roles in mechanical response, inflammation, metabolism, drug treatment delivery and so on (Yuan et al. 2017; Lee et al. 2019; Ning et al. 2019; Hegde et al. 2023). Among them, micro RNAs (miRNAs) are such kinds of noncoding RNAs that share unique abilities to modulate multiple protein networks simultaneously. Moreover, miRNAs have attracted attention and popularity in clinically oriented research because they can be easily manufactured into drug delivery formulations. Additionally, mechanosensitive miRNAs like miR‐155‐5p, which regulates downstream genes involved in inflammation in intervertebral disc (IVD) cells in IVD degeneration (Cazzanelli et al. 2024), while silencing of miR‐138‐5p sensitizes bone anabolic action to mechanical stimuli in vivo (Chen et al. 2020). Nevertheless, the specific involvement and therapeutic potential of miRNAs in the context of mechanical overload‐induced degeneration of cartilage and subchondral bone during OA progression remain poorly characterized.

By utilizing synovium fluids from 96 temporomandibular disorders (TMD) patients, our study identified that miR‐330 is closely related to OA progression. Further OA clinical specimens and animal models revealed miR‐330 was dysregulated during both abnormal mechanical stresses. As a key regulatory molecule for multiple system diseases, miR‐330 has been reported to have an expression level that is highly correlated with the occurrence, development, and prognosis of diseases such as tumors, cardiovascular diseases, and neurological disorders (Mubaid et al. 2019). However, its role in osteoarthritis (OA) has not yet been clarified. Thus, we further investigated its critical role in OA progression using gene‐edited mice.

## 2 Results

### 2.1 miR‐330 Is a Mechanical Responsive Micro RNA That Correlates With Osteoarthritis Progression

**FIGURE 1:**
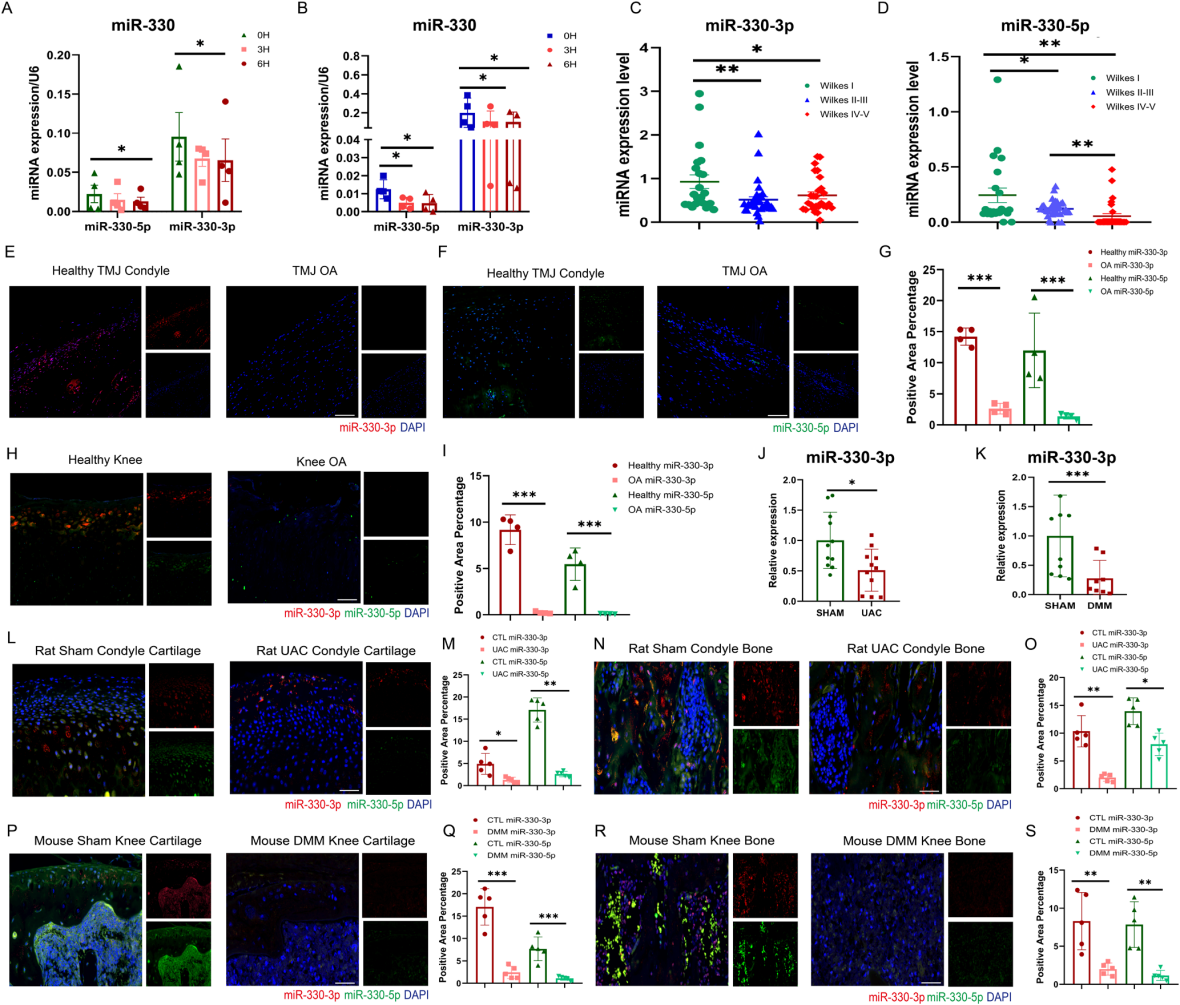
MiR‐330 was downregulated in mechanical stress induced OA progress in both clinical specimen as well as in vivo and in vitro models. (A) RT‐qPCR comparison of the miR‐330‐3p and miR‐330‐5p under mechanical stimulation in murine chondrocytes (*n* = 4). (B) RT‐qPCR comparison of the miR‐330‐3p and miR‐330‐5p under mechanical stimulation in murine BMMs (*n* = 4). (C) The expression of miR‐330‐3p in different stages of the clinical joint fluid samples (*n* = 84). (D) The expression of miR‐330‐5p in different stages of the clinical joint fluid samples (*n* = 84). (E) FISH staining of miR‐330‐3p (red) in the condylar cartilage subchondral bone samples of clinical specimen, scale bar = 100 μm (*n* = 4). (F) FISH staining of miR‐330‐5p (green) in condylar bone‐cartilage samples of clinical samples, scale bar = 100 μm (*n* = 4). (G) Statistical analysis of fluorescent intensity of Figure E‐F, showed miR‐330‐3p and miR‐330‐5p were significantly down regulated in human TMJ OA cartilage and subchondral bone. (H) FISH staining of miR‐330‐3p (red) and miR‐330‐5p (green) in condylar bone‐cartilage samples of clinical samples, scale bar = 50 μm (*n* = 4). (I) Statistical analysis of fluorescent intensity of Figure H, showed miR‐330‐3p/5p was significantly down regulated in human knee OA cartilage and subchondral bone. (J) RT‐qPCR test of miR‐330‐3p expression in rat cartilage and subchondral bone tissue in TMJ OA group as well as unmodeled group (*n* = 11 or 12). (K) RT‐qPCR test of miR‐330‐3p expression in mice cartilage and subchondral bone tissue in DMM leading knee OA group as well as unmodeled group (*n* = 10 or 8). (L) FISH staining of miR‐330‐3p (red) and miR‐330‐5p (green) in condylar cartilage tissue of rats in both UAC leading OA and control group, scale bar = 50 μm (*n* = 5). (M) Statistical analysis of Figure L, showed miR‐330‐3p/5p were both significantly depressed in rat UAC leading TMJOA model cartilage. (N) FISH staining of miR‐330‐3p (red) and miR‐330‐5p (green) in condylar subchondral bone tissue of rats in both UAC leading OA and control group, scale bar = 50 μm (*n* = 5). (O) Statistical analysis of Figure N, showed miR‐330‐3p/5p were both significantly depressed in rat UAC leading TMJOA model subchondral bone. (P) FISH staining of miR‐330‐3p (red) and miR‐330‐5p (green) in knee cartilage tissue of rats in both DMM leading OA and control group, scale bar = 50 μm (*n* = 5). (Q) Statistical analysis of Figure P, showed miR‐330‐3p/5p were both significantly depressed in mouse DMM leading knee OA model cartilage. (R) FISH staining of miR‐330‐3p (red) and miR‐330‐5p (green) in knee subchondral bone tissue of rats in both DMM leading OA and control group, scale bar = 50 μm (*n* = 5). (S) Statistical analysis of Figure R, showed miR‐330‐3p/5p were both significantly depressed in mouse DMM leading knee OA model subchondral bone. The error bar represents the mean ± SD. ns, nonsignificant. *p < 0.05, **p < 0.01, ***p < 0.001 by unpaired Student's *t*‐test.

To investigate miRNAs that are closely related to mechanical response and OA progression, we first performed miRNA sequencing on synovial fluid from 12 temporomandibular disorders (TMD) patients based on radiological diagnosis and magnetic resonance imaging (MRI) grouping based on Wilkes stages classification (Figure S1A–C). There were 65 differentially expressed miRNAs (DEMs) detected, with 16 upregulated in OA progression and 49 downregulated. In addition, differently expressed miRNAs in unilateral anterior crossbite (UAC)‐induced rat TMJOA models found 106 miRNAs, with 14 upregulated and 92 downregulated (Figure S1D,E). Thirteen miRNAs showed consistent upregulation or downregulation in both human and animal specimen sequencing datasets (Figure S1F). Subsequent validation by real‐time quantitative polymerase chain reaction (RT‐qPCR) in mechanical overloading primary chondrocytes and bone marrow‐derived macrophages (BMMs) showed that there were 6 mechanical sensitive miRNAs in chondrocytes and 9 in BMMs (Figure S1G,H, Figure 1A,B), and 5 were the same in the two cells. Notably, among them only miR‐330‐3p exhibited pronounced expression changes following progressing mechanical stimulation in vitro, so as the mature miR‐330‐5p from the other site (Figure 1A,B). RT‐qPCR analysis of patient TMJOA synovial fluid and condylar samples confirmed significant downregulation of both miR‐330‐3p and miR‐330‐5p compared to controls (Figure 1C,D, Table.S1). Receiver operating characteristic (ROC) curve analysis further demonstrated that miR‐330‐3p showed 76.88% of area under curve (AUC) of ROC analysis, and miR‐330‐5p displayed 64.83% AUC for diagnosis, which both showed positive diagnostic value for TMJOA progression (Figure S2A,B). Fluorescence in situ hybridization (FISH) confirmed reduced expression of both miRNAs in OA‐affected cartilage and subchondral bone of TMJ (Figure 1E–G) and knee joints (Figure 1H,I).

Consistent with human TMJ fluid observations, rat condylar cartilage and subchondral bone tissue analysis by RT‐qPCR showed much more abundant expression of miR‐330‐3p but not miR‐330‐5p (Figure S2C). Both UAC‐induced rat TMJOA and destabilization of the medial meniscus (DMM)‐induced mouse knee OA models demonstrated significantly decreased miR‐330‐3p concentrations under abnormal mechanical loading (Figure 1J,K). FISH analysis further confirmed progressive downregulation of miR‐330‐3p in cartilage and subchondral bone throughout OA progression in mechanically unstable TMJ and knee joint models (Figure 1L–S, Figure S2D–G).

Collectively, these findings demonstrate that mechanical stress significantly downregulates miR‐330‐3p expression, with progressive reduction occurring during OA advancement across patients and animal models.

### 2.2 Deficiency of miR‐330 in Cartilage or Macrophages Results in Cartilage Degeneration and Bone Loss Phenotype

**FIGURE 2:**
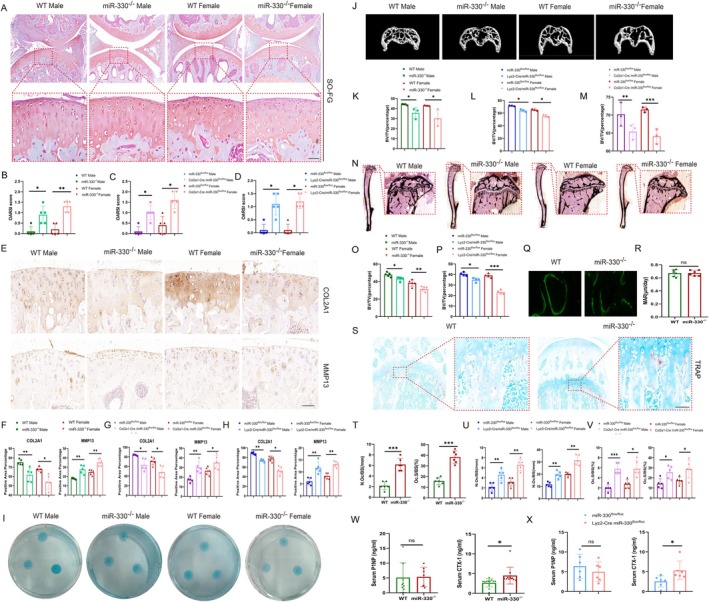
Comparison of physiological phenotypes of osteochondral tissue in WT mice and miR‐330^−/−^ mice. (A) Typical figures showed the comparison of physiologic SO staining of knee joints of WT mice and miR‐330^−/−^ mice, scale bar = 50 μm (*n* = 5). (B) The statistical analysis of OARSI score of physiologic WT mice and miR‐330^−/−^ mice knee joints. (C) The statistical analysis of OARSI score of physiologic miR330^flox/flox^ mice and miR330^flox/flox^Col2a1‐cre^ERT^ mice knee joints. (D) The statistical analysis of OARSI score of physiologic miR330^flox/flox^ mice and miR330^flox/flox^Lyz2‐cre mice knee joints. (E) IHC staining of knee cartilage of WT and miR‐330^−/−^ mice for MMP13 and COL2A1, scale bar = 50 μm (*n* = 5). (F) The statistical analysis of IHC positive rate of physiologic WT mice and miR‐330^−/−^ mice knee joints with MMP13 and COL2A1 staining. (G) The statistical analysis of IHC positive rate of physiologic miR330^flox/flox^ mice and miR330^flox/flox^Col2a1‐cre^ERT^ mice knee joints with MMP13 and COL2A1 staining. (H) The statistical analysis of IHC positive rate of physiologic miR330^flox/flox^ mice and miR330^flox/flox^Lyz2‐cre mice knee joints with MMP13 and COL2A1 staining. (I) Micro mass culture with Alisin Blue staining of chondrocytes from of WT mice and miR‐330^−/−^ mice (*n* = 3). (J) Micro CT detection of knee joints subchondral bone area of WT mice and miR‐330^−/−^ mice in phenotype (*n* = 3 or 4). (K) The statistic of bone mass analysis of subchondral bone area from WT mice and miR‐330^−/−^ mice in phenotype. (L) The statistic of bone mass analysis of subchondral bone area from miR330^flox/flox^ mice and miR330^flox/flox^Lyz2‐cre mice knee joints in phenotype. (M) The statistic of bone mass analysis of subchondral bone area from miR330^flox/flox^ mice and miR330^flox/flox^Col2a1‐cre^ERT^ mice knee joints in phenotype. (N) Von‐Kossa staining of hard tissue sections of knee joints of WT mice and miR‐330^−/−^ mice, scale bar = 400 μm (*n* = 5). (O) The statistic of subchondral bone mass analysis of Van‐Kossa staining from WT mice and miR‐330^−/−^ mice in phenotype. (P) The statistic of subchondral bone mass analysis of Van‐Kossa staining from miR330^flox/flox^ mice and miR330^flox/flox^Lyz2‐cre mice in phenotype. (Q) Calcein AM labeling osteogenesis in the subchondral bone of knee joints of WT mice and miR‐330^−/−^ mice (*n* = 6). (R) The statistical analysis of bone formation rate in subchondral area of knee from WT mice and miR‐330^−/−^ mice. (S) TRAP staining of knee joints of WT mice and miR‐330^−/−^ mice in physiological conditions, scale bar = 50 μm (*n* = 6). (T) Statistical analysis of in vivo TRAP positive rate in subchondral area of WT mice and miR‐330^−/−^ mice knee joints in phenotype. (U) Statistical analysis of in vivo TRAP positive rate in subchondral area of miR330^flox/flox^ mice and miR330^flox/flox^Lyz2‐cre mice knee joints in phenotype. (V) Statistical analysis of in vivo TRAP positive rate in subchondral area of miR330^flox/flox^ mice and miR330^flox/flox^Col2a1‐cre^ERT^ mice joints in phenotype. (W) P1NP and CTX‐1 concentration in serum of WT mice and miR‐330^−/−^ mice determined by ELISA (*n* = 8 or 12). (X) P1NP and CTX‐1 concentration in serum of miR330^flox/flox^ mice and miR330^flox/flox^Lyz2‐cre mice tested by ELISA (*n* = 6). The error bar represents the mean ± SD. ns, nonsignificant. *p < 0.05, **p < 0.01, ***p < 0.001 by unpaired Student's *t*‐test.

To further investigate miR‐330's role in OA‐related subchondral bone and cartilage degeneration, we generated gene‐edited mice with miR‐330 global knock out (miR‐330^−/−^ mice), and conditional knock out of Col2a1‐cre^ERT2^ miR‐330^flox/flox^ mice in chondrocytes and Lyz2‐cre miR‐330^flox/flox^ mice in osteoclast for further research. At 12 weeks, Safranin O (SO) staining revealed exacerbated cartilage degeneration in both sexes of miR‐330 global knock out mice, with significantly higher OARSI scores (Figure 2A,B). Both conditional knock out in chondrocytes and osteoclasts exhibited reduced SO staining intensity compared to their controls (Figure S3A,B, Figure 2C,D). Immunohistochemistry (IHC) staining showed decreased COL2A1 and increased MMP13 expression in miR‐330 global knock out mice cartilage (Figure 2E,F), corroborated in conditional knockouts (Figure S3C,D, Figure 2G,H). In vitro, primary miR‐330 global knock out chondrocytes demonstrated impaired chondrogenic differentiation in 10‐day micro mass induction cultures versus WT chondrocytes (Figure 2I). TUNEL staining revealed increased chondrocytes apoptosis in vivo and in vitro (Figure S3E–H), and further confirmed by flow cytometric Annexin V/PI analysis in vitro (Figure S3I,J).

Micro‐CT analysis of 12‐week‐old miR‐330 global knock out mice showed reduced femoral cancellous bone volume/tissue volume (BV/TV) ratio, decreased trabecular number (Tb.N), and increased trabecular separation (Tb.Sp) versus WT mice (Figure S4A,B). Similar trends were found in mice conditional knock out in osteoclasts as well (Figure S4C,D). Given the clinical relevance of subchondral bone changes, we observed significantly lower BV/TV ratio in miR‐330 global knock out mice knee subchondral bone (Figure 2J,K) and conditional knock out in osteoclasts (Figure S4E, Figure 2L). Cartilage‐specific miR‐330 deletion also reduced subchondral bone mineral density and BV/TV ratio as well (Figure S4F, Figure 2M). Histomorphormetry analysis with Von Kossa staining confirmed decreased BV/TV in both miR‐330 global knock out and osteoclast conditional knock out subchondral bone (Figure 2N–P, Figure S4G).

Our analysis revealed miR‐330 knockout does not affect osteoblast function, including fluorescence dual label osteogenesis in vivo (Figure S4H,I, Figure 2Q,R) and cellular osteogenic induction in vitro (Figure S4J). Furthermore, miR‐330 knockout enhanced osteoclast differentiation, evidenced by enhanced osteoclast number in subchondral bone in vivo (Figure 2S,T), and the same trend was also found in both conditional knockout in osteoclasts and chondrocytes in vivo (Figure S4K,L, Figure 2U–V). Besides, serum P1NP content in miR‐330 global knockout and conditional knockout in osteoclasts in vivo showed no significant difference which further confirmed the non‐significant impact in bone formation. While, the upregulation of serum CTX‐I level supports the above findings in osteoclast formation (Figure 2W,X). The in vitro experiment also confirmed that miR‐330 knockout enhances the TRAP staining area and number of induced osteoclasts (Figure S4M,N). It can be addressed that the downregulation of miR‐330 could mainly regulate the chondrocytes degeneration and osteoclast formation in vivo and in vitro.

### 2.3 miR‐330 Is Critical in Mitigating Cartilage Degeneration and Bone Loss in OA

**FIGURE 3:**
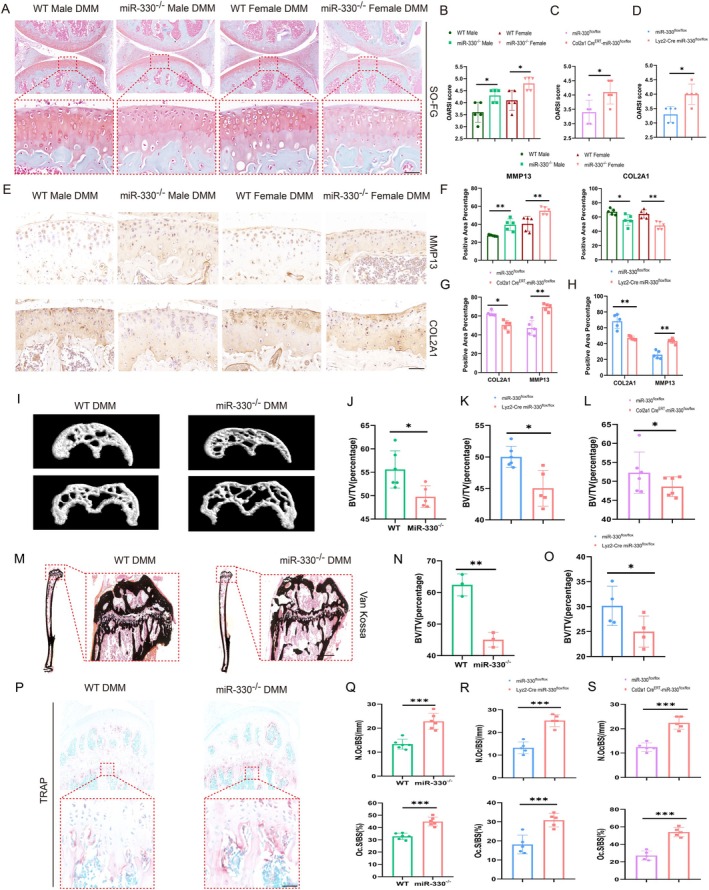
Comparison of osteochondral tissue pathological phenotype induced by abnormal mechanical stress in WT mice and miR‐330^−/−^ mice. (A) Comparison of SO staining between WT mice and miR‐330^−/−^ mice knee under DMM‐induced OA conditions, scale bar = 50 μm (*n* = 5). (B) The statistical analysis of OARSI score of DMM modeled WT mice and miR‐330^−/−^ mice knee joints. (C) The statistical analysis of OARSI score of DMM modeled miR330^flox/flox^ mice and miR330^flox/flox^Col2a1‐cre^ERT^ mice knee joints. (D) The statistical analysis of OARSI score of DMM modeled miR330^flox/flox^ mice and miR330^flox/flox^Lyz2‐cre mice knee joints. (E) IHC staining of COL2A1 and MMP13 in knee cartilage of WT mice and miR‐330^−/−^ mice under DMM‐induced OA conditions, scale bar = 50 μm (*n* = 5). (F) The statistical analysis of IHC positive rate of DMM modeled OA WT mice and miR‐330^−/−^ mice knee joints with MMP13 and COL2A1 staining. (G) The statistical analysis of IHC positive rate of DMM modeled OA miR330^flox/flox^ mice and miR330^flox/flox^Col2a1‐cre^ERT^ mice knee joints with MMP13 and COL2A1 staining. (H) The statistical analysis of IHC positive rate of DMM modeled OA miR330^flox/flox^ mice and miR330^flox/flox^Lyz2‐cre mice knee joints with MMP13 and COL2A1 staining. (I) Micro CT detection of knee joints subchondral bone area of WT mice and miR‐330^−/−^ mice under DMM knee OA model (*n* = 6). (J) The statistic of bone mass analysis of subchondral bone area from WT mice and miR‐330^−/−^ mice under DMM knee OA model. (K) The statistic of bone mass analysis of subchondral bone area from miR330^flox/flox^ mice and miR330^flox/flox^Lyz2‐cre mice knee joints under DMM knee OA model. (L) The statistic of bone mass analysis of subchondral bone area from miR330^flox/flox^ mice and miR330^flox/flox^Col2a1‐cre^ERT^ mice knee joints under DMM knee OA model. (M) Von‐Kossa staining of hard tissue sections of OA knee of WT mice with miR‐330^−/−^ mice, scale bar = 400 μm (*n* = 3). (N) The statistic of subchondral bone mass analysis of Van‐Kossa staining from WT mice and miR‐330^−/−^ mice in DMM leading OA model. (O) The statistic of subchondral bone mass analysis of Van‐Kossa staining from miR330^flox/flox^ mice and miR330^flox/flox^Lyz2‐cre mice in DMM leading OA model. (P) TRAP staining and statistical analysis of DMM induced knee OA from both WT mice and miR‐330^−/−^ mice, scale bar = 50 μm (*n* = 6). (Q) Statistical analysis of in vivo TRAP positive rate in subchondral area of WT mice and miR‐330^−/−^ mice DMM induced knee OA joints. (R) Statistical analysis of in vivo TRAP positive rate in subchondral area of miR330^flox/flox^ mice and miR330^flox/flox^Lyz2‐cre mice DMM induced knee OA joints. (S) Statistical analysis of in vivo TRAP positive rate in subchondral area of miR330^flox/flox^ mice and miR330^flox/flox^Col2a1‐cre^ERT^ mice DMM induced knee OA joints. The error bar represents the mean ± SD. ns, nonsignificant. *p < 0.05, **p < 0.01, ***p < 0.001 by unpaired Student's *t*‐test.

Both global knockout and conditional knockout of miR‐330 in either chondrocytes or osteoclasts exhibited reduced Safranin O (SO) staining intensity and elevated OARSI scores in DMM induced knee OA model, indicating exacerbated cartilage degeneration under abnormal mechanical loading (Figure 3A–D; Figure S5A,B). These models additionally demonstrated decreased COL2A1 expression and increased MMP13 levels in articular cartilage in IHC staining, which indicates the increase of catabolism in chondrocytes without miR‐330 protection (Figure 3E–H; Figure S5C,D). Apoptosis rates were also significantly elevated across miR‐330 knockout with DMM‐induced knee OA in vivo (Figure S5E,F). In vitro, mechanical compression and TNF‐α stimulation both induced substantially higher apoptosis rates in miR‐330‐deficient primary chondrocytes (Figure S5G–J).

Micro‐CT evaluation of subchondral bone remodeling post‐OA induction revealed significantly reduced BV/TV ratio and more severe bone loss in miR‐330 global and conditional knockout mice in chondrocytes and osteoclasts versus respective controls (Figure 3I–L, Figure S6A,B). These phenomena were corroborated by Von Kossa staining, confirming enhanced subchondral bone loss following miR‐330 deletion in both global and osteoclast specific conditional knockout animal models (Figure 3M–O; Figure S6C). TRAP staining analysis further demonstrated that genetic ablation of miR‐330, no matter global or tissue‐specific in osteoclast lineage, both promoted osteoclast activation in DMM‐induced knee OA mice model (Figure 3P–S; Figure S6D,E). The above evidence further demonstrated the key protective function of miR‐330 in abnormal mechanical loading induced OA progression in preserving cartilage and subchondral bone disorder and degeneration.

### 2.4 miR‐330 Can Balance Cartilage‐Subchondral‐Bone Stability by Directly Regulating Genes Including CTGF, FGFR1, EPOR, IL‐1β and TNF‐α

**FIGURE 4:**
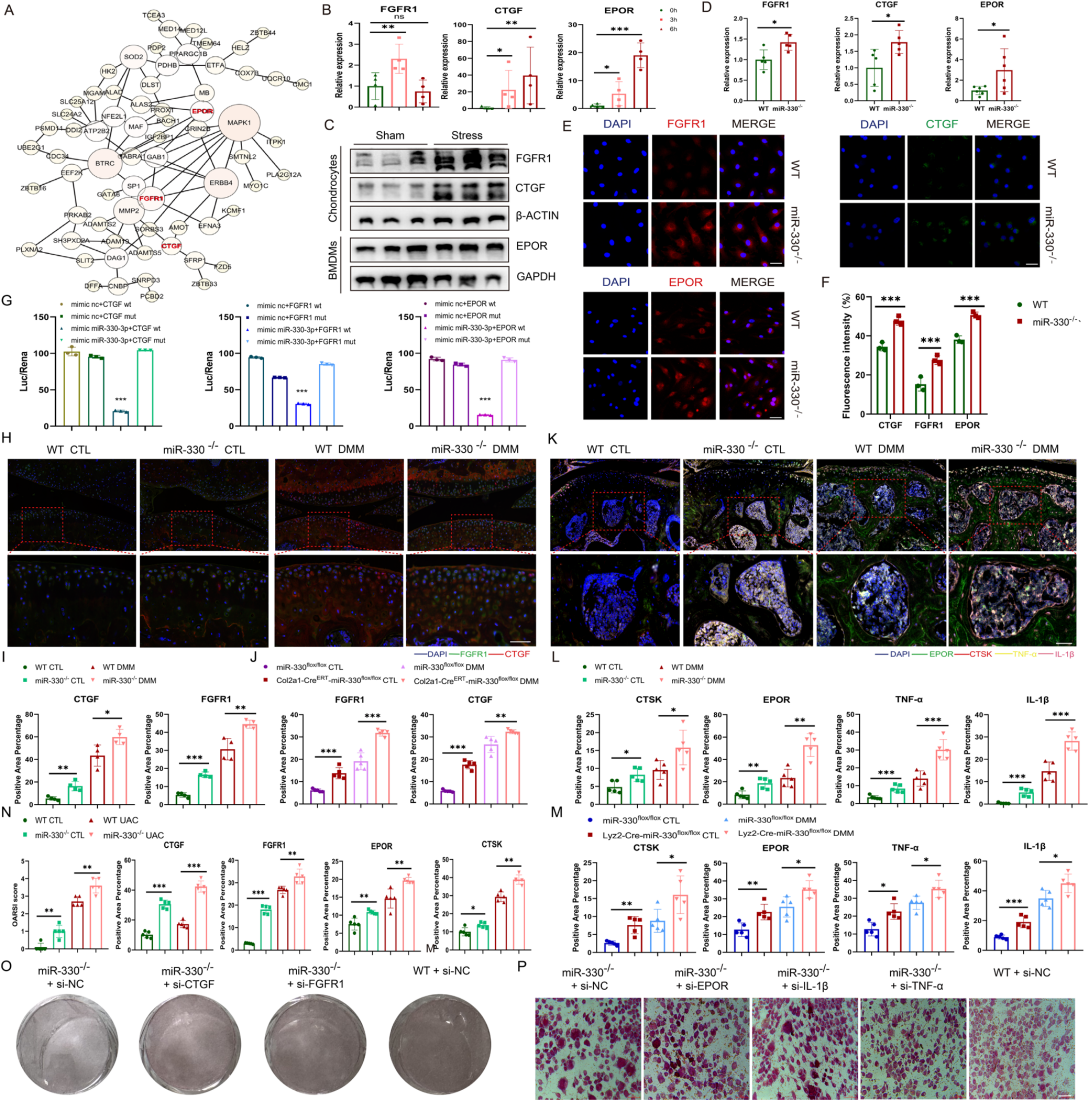
The function and regulation of targeted genes by miR‐330 in cartilage. (A) Predicted interactions of the proteins encoded by the intersecting partial differential expressed genes from the cartilage and BMMs RNA sequencing and the selected key targeted genes (red). (B) RT‐qPCR assay of the expression of FGFR1, CTGF under mechanical stress stimulation in chondrocytes and EPOR in BMMs (*n* = 4). (C) Western Blot assay of the expression of FGFR1, CTGF under mechanical stress stimulation in chondrocytes and EPOR in BMMs (*n* = 3). (D) RT‐qPCR assay of the expression of FGFR1 and CTGF in primary chondrocytes, EPOR in BMMs from WT and miR‐330^−/−^ mice (*n* = 5). (E, F) IF staining and the statistical analysis of fluorescence intensity of FGFR1 and CTGF in primary chondrocytes, EPOR in BMMs from WT mice and miR‐330^−/−^ mice, scale bar = 100 μm (*n* = 3). (G) Luciferase assay to confirm the direct binding relationship of miR‐330‐3p with CTGF, FGFR1 and EPOR (*n* = 3). (H) IF staining to detect the expression changes of CTGF and FGFR1 in the knee of physiological and DMM‐induced OA condition in WT mice and miR‐330^−/−^ mice, scale bar = 50 μm (*n* = 4). (I) Statistical analysis of IF staining of FGFR1 and CTGF in WT mice and miR‐330^−/−^ mice knee physiological and DMM‐induced OA condition. (J) Statistical analysis of IF staining of FGFR1 and CTGF in miR330^flox/flox^ mice and miR330^flox/flox^Col2a1‐cre^ERT^ mice knee physiological and DMM‐induced OA condition. (K) IF staining to detect the expression changes of EPOR, TNF‐α, IL‐1β in the knee of physiological and DMM‐induced OA condition in WT mice and miR‐330^−/−^ mice, scale bar = 50 μm (*n* = 5). (L) Statistical analysis of IF staining of EPOR, TNF‐α, IL‐1β in WT mice and miR‐330^−/−^ mice knee physiological and DMM‐induced OA condition. (M) Statistical analysis of IF staining of EPOR, TNF‐α, IL‐1β in miR330^flox/flox^ mice and miR330^flox/flox^Lyz2‐cre mice knee physiological and DMM‐induced OA condition. (N) Statistical analysis of IHC staining of CTGF, FGFR1, EPOR and CTSK in WT mice and miR‐330^−/−^ mice TMJ physiological and UAC‐induced OA condition. (O) SO staining comparison of chondrocytes from miR‐330^−/−^ mice and WT mice after targeted interference with FGFR1 and CTGF (*n* = 3). (P) TRAP staining comparison of BMMs induced osteoclast from miR‐330^−/−^ mice and WT mice after targeted interference with EPOR, TNF‐α and IL‐1β, scale bar = 100 μm (*n* = 3). The error bar represents the mean ± SD. ns, nonsignificant. *p < 0.05, **p < 0.01, ***p < 0.001 by unpaired Student's *t*‐test.

In order to explore the underlying mechanisms on how miR‐330 regulates chondrocytes and osteoclasts hemostasis, we utilized bioinformatic analysis of cartilage RNA sequencing data from miR‐330^−/−^ mice and identified 1820 differential expressed genes (DEGs) with more than 2‐fold changes versus WT mice (Figure S7A,B). Intersection of upregulated DEGs with TargetScan/ENCORI‐predicted targets of miR‐330 revealed 73 high‐confidence targets genes (Figure S7C). GO and KEGG enrichment analyses demonstrated that miR‐330 ablation promotes cartilage catabolism activation, angiogenesis formation, oxidative stress upregulation, and osteoclast differentiation (Figure S7D,E). Parallel RNA sequencing of miR‐330^−/−^ osteoclasts compared with WT identified 42 upregulated putative targets among 2200 DEGs in the two databases (Figure S7F–J). Furthermore, we compared these genes with the differentially expressed proteins from TMJOA synovium fluids protein mass spectrometry with protein–protein interactions (PPI) analysis. Interestingly, we finally identified CTGF, FGFR1 and EPOR as targeting genes that not only changed significantly during patients TMJOA development but also predicted as the direct miR‐330 target genes (Figure 4A, Figure S8A–E). Besides, based on our previous study, the key inflammatory factors as TNF‐α and IL‐1β were also predicted as the directed target genes of miR‐330 in OA.

Further RT‐qPCR and western blot results showed that the expression of CTGF, FGFR1, and EPOR is significantly upregulated by mechanical stress loading (Figure 4B,C), which was in accordance with the down‐regulation of miR‐330 under mechanical overloading. Next, we performed RT‐qPCR of primary chondrocytes and BMMs gained from miR‐330 global knockout mice and WT mice, and the results showed that the mRNA expression of CTGF, FGFR1, and EPOR was all significantly elevated (Figure 4D, Figure S8F). Further immunofluorescence (IF) staining of chondrocytes and BMMs showed higher protein expression levels of CTGF, FGFR1, and EPOR in vitro with miR‐330 knockout (Figure 4E,F). The dual‐luciferase reporter assays revealed that miR‐330‐3p can directly regulate CTGF, FGFR1, and EPOR mRNA degradation (Figure 4G, Figure S8H). Multiplex IHC staining with tyramide signal amplification (TSA) labeling of mouse knee joint showed that CTGF and FGFR1 were significantly upregulated in miR‐330 global knockout mice cartilage tissue under both physiological and OA conditions (Figure 4H,I). The miR‐330 targeted gene changes in cartilage were further verified in Col2a1‐CKO mice which displayed the same trend (Figure S9A, Figure 4J). TSA labeled IHC staining of mouse knee joint under both physiological and OA conditions showed that EPOR was significantly upregulated in CTSK‐positive osteoclasts after miR‐330 global knockout (Figure 4K,L) and osteoclast conditional knockout mice as well (Figure S9B, Figure 4M). Furthermore, the severer degeneration of cartilage and subchondral bone in TMJOA progression as well as the above‐mentioned upregulated target genes with miR‐330 global knockout mice was further proved by UAC induced TMJOA model in vivo (Figure 4N, Figure S9C).

To further verify the function of targeted genes of miR‐330 in chondrocytes, it was first proved that after FGFR1 and CTGF targeting knockdown in miR‐330 global knockout mice primary chondrocytes (Figure S10A), the SO staining intensity increased (Figure 4O) and the apoptosis ratio was significantly reduced in vitro (Figure S10B,C). On the other hand, TRAP staining of osteoclasts is attenuated after targeted knockdown of EPOR, IL‐1β, and TNF‐α in miR‐330 global knockout mice macrophages (Figure S10A, Figure 4P). Overall, miR‐330 could affect cartilage homeostasis not only by directly regulating CTGF and FGFR1 in chondrocytes, but also with impact from subchondral bone regulation via EPOR, IL‐1β, and TNF‐α.

### 2.5 Injection of miR‐330 AAV Mitigated the Degeneration of the Cartilage and Subchondral Bone

**FIGURE 5:**
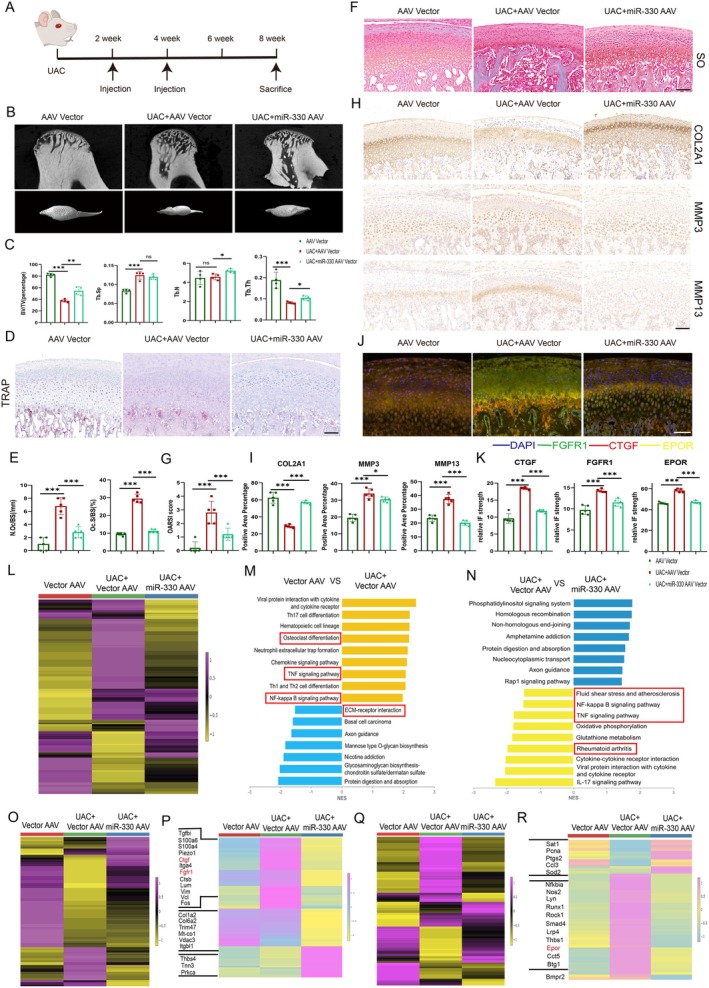
The intra‐articular injection of miR‐330 AAV for TMJ OA treatment and the single‐cell RNA seq analysis. (A) Model establishment diagram of the experimental flow of UAC and articular injection in rats. (B, C) Micro CT of condyles from UAC modeling rats intra‐articular injected with Vector AAV and miR‐330 AAV as well as AAV injected sham group and the statistical analysis of bone mass (*n* = 4). (D) TRAP staining after sectioning of condyles of UAC modeling rats intra‐articular injected with Vector AAV and miR‐330 AAV as well as AAV injected sham group, scale bar = 100 μm (*n* = 5). (E) The statistics of positive TRAP staining changes with or without miR‐330 AAV injection of TMJOA. (F) SO staining after sectioning of condyles of UAC modeling rats intra‐articular injected with Vector AAV and miR‐330 AAV as well as AAV injected sham group, scale bar = 100 μm (*n* = 5). (G) The statistics of OARSI score changes with or without miR‐330 AAV injection of TMJOA. (H) IHC staining of MMP13, MMP3 and COL2A1 after UAC modeling rats intra‐articular injected with Vector AAV and miR‐330 AAV as well as AAV injected sham group (*n* = 5). (I) The statistics of IHC positive rate changes of MMP13, MMP3 and COL2A1 in slices with or without miR‐330 AAV injection of TMJOA as well as AAV injected sham group. (J) IHC staining with TSA labeling of miR‐330 target genes EPOR, FGFR1 and CTGF after UAC modeling rats intra‐articular injected with Vector AAV and miR‐330 AAV as well as AAV injected sham group (*n* = 5). (K) The statistics of IHC positive rate changes of target genes EPOR, FGFR1 and CTGF in slices with or without miR‐330 AAV injection of TMJOA as well as AAV injected sham group. (L) Heatmap of single‐cell RNA sequencing of gene expression in cartilage‐subchondral bone tissue of condylar eminence of UAC‐modeled rats with intra‐articular injections of Vector AAV or miR‐330 AAV and the unmodeled rats. (M) GSEA analysis of genes in UAC modeled and unmodeled rats injected with Vector AAV. (N) GSEA analysis of genes in UAC modeled rats injected with miR‐330 AAV vs. UAC modeled rats injected with Vector AAV. (O) Heat map of differential gene expression in chondrocyte cluster of UAC‐modeled rats and unmodeled rats injected with Vector AAV or miR‐330 AAV. (P) Heat map of differential gene expression regulated by or co‐interacted with FGFR1 and CTGF in chondrocyte populations. (Q) Heatmap of differential genes in macrophage‐osteoclast populations of UAC modeled rats versus control rats with articular injection of Vector AAV and miR‐330 AAV. (R) Heat map of differential gene expression regulated by or co‐interacted with EPOR, IL‐1β and TNF‐α in macrophage‐osteoclast populations. The error bar represents the mean ± SD. ns, nonsignificant. *p < 0.05, **p < 0.01, ***p < 0.001 by unpaired Student's *t*‐test.

The aforementioned evidence suggested mir‐330 as a potential therapeutic miRNA for the treatment of OA, therefore we injected miR‐330 AAV into joint under UAC model (Figure 5A, Figure S11A,B). Compared with the AAV vector injection group, the results of FISH staining proved that the expression of miR‐330 was significantly increased in the cartilage and subchondral bone of the miR‐330 AAV injection group (Figure S11C,D). Micro CT showed increased bone mineral density in the treatment group (Figure 5B,C). The positive rate of osteoclast TRAP staining was significantly reduced and SO staining intensity was obviously increased (Figure 5D–G). IHC staining showed that the expression of COL2A1 was up‐regulated, while the expression of MMP3 and MMP13 was significantly reduced after miR‐330 AAV treatment. Besides, the decrease of miR‐330 targeting genes such as CTGF, FGFR1, and EPOR in cartilage and subchondral bone tissue was also proved by TSA labeled IHC (Figure 5H–K). Single‐cell RNA (sc‐RNA) sequencing was further carried out to prove the miR‐330 supplement regulation function via analysis of intraarticular AAV injection rat condyle cartilage and subchondral bone tissue (Figure S12A–C). The overall heatmap and GSEA analysis showed that osteoclast formation, TNF‐α pathway, and NF‐κB pathway were activated in the condylar cartilage and subchondral bone of UAC induced TMJOA rats. After miR‐330 AAV injection, the activation of the NF‐κB pathway as well as fluid shear stress sensitivity were inhibited (Figure 5 L‐N). After the cell cluster was defined, it showed that the normal ratios of condylar chondrocytes, osteoblasts, and osteoclasts were restored after miR‐330 AAV injection (Figure S12D,E). Also, significant gene expression changes and cell heterogeneity with UAC establishment as well as miR‐330 AAV treatment were found which further corroborates the above results (Figure S12F–H). Based on the cell clusters, it can be found that FGFR1 and CTGF are mainly expressed in cartilage tissue (Figure S12I,J). With the analysis of chondrocyte changes, it was found that miR‐330 AAV injection can alleviate the catabolism of chondrocytes, mitigate the effects of mechanical stimulation on cells, and promote cartilage repair, and the main effectiveness could be impacted by the target genes such as FGFR1 and CTGF. The extracellular matrix organization and mechanical stimulation feedback could be healthily regulated (Figure 5O,P, Figure S13A–D, Figure S14A,B). While EPOR, TNF‐α, and IL‐1β are mainly expressed in macrophage‐osteoclasts (Figure S12I,K), and miR‐330 significantly inhibits the activation of IL‐17, NF‐κB, and TNF‐α signaling pathways active in macrophage‐osteoclasts, the secretion and production of cytokines were heavily controlled (Figure 5Q,R, Figure S13E–H, Figure S14C,D), which strongly supports the previous RNA‐seq results of osteoclasts from miR‐330 global knockout mice compared with the WT mice. In osteogenesis‐related cells, miR‐330 can regulate the overactivation of the HIF‐1α pathway and the level of glucose metabolism, thereby protecting bone tissue as well (Figure S13I,K). Through sc‐RNA sequencing and analysis after miR‐330 AAV intraarticular injection, we can further confirm the conclusions of miR‐330 global knockout mice RNA sequencing, that miR‐330 can protect cartilage and subchondral bone from degeneration in OA by affecting the catabolic metabolism and mechanical response performance of both chondrocytes and osteoclasts in vivo.

## 3 Discussion

Osteoarthritis (OA) is a degenerative joint disease involving multiple articular tissues, including bone, cartilage, and synovium. Although several studies have demonstrated its strong association with aberrant mechanical stress‐induced pathological changes, mechanosensitive microRNAs as the key regulator implicated in both knee OA and TMJOA remain insufficiently identified. It has been reported that mechanical overloading‐induced miR‐325‐3p reduction can promote chondrocyte senescence in facet joint (Zhao et al. 2023). Besides, miR‐3085‐3p as a modulator of cartilage degeneration in facet joint osteoarthritis (Lai et al. 2025). While the underlying mechanism and the therapeutic effect of these micro RNAs were insufficiently explored and proved. Mechanosensitive micro RNAs regulated by anabolic and catabolic loading were first reported by Hecht et al. 2019, but it focused more on the in vitro simulation while neglecting the in vivo evidence exploration (Hecht et al. 2019). Shang Xiaobin has summarized the micro RNAs that may have a mechanical response in OA development progression and tentative treatment attempts, but miR‐330 is not among them and has not yet been confirmed or reported previously (Shang 2025). In this study, we first investigated the possible key miRNAs in abnormal mechanical stress‐induced OA via micro tissue RNA sequencing in human and animal models and in vitro experiment confirmation. There were 13 micro RNAs identified with statistical differences, among them 8 were reported previously with mechanical sensitivity, and the other 5 were first discovered. miR‐330‐3p is expressed with the highest abundance among the 5 first reported mechanical sensitive micro RNAs. Analysis of synovial fluid from 96 patients in different Wilkes stages combined with FISH staining of mandibular condyles revealed significant downregulation of miR‐330 in both early‐ and late‐stage OA. Subsequent animal experiments demonstrated that miR‐330 deficiency exacerbates bone and cartilage degeneration related to aggravated catabolism in both physiological and pathological models induced by abnormal mechanical stress with gene‐edited miR‐330 global knock out and conditional knock out mice. Our study could address the point that compared with miR‐330‐5p, miR‐330‐3p showed higher abundance in both chondrocytes and the macrophage‐osteoclast system, which mainly impacts cartilage and subchondral bone in OA with mechanical stimulation feedback. Bio‐informatic analysis showed that miR‐330‐3p regulates both cartilage catabolism and osteoclast formation and miR‐330‐5p plays an important role in regulating the production of IL‐1β and the interaction of cytokine secretion as well. In contrast to their distinct functions in cardiovascular‐related studies (Wei et al. 2019; Zuo et al. 2021; Shan et al. 2023), miR‐330‐3p and miR‐330‐5p are both beneficial to OA reversing verified by the supplement of miR‐330 in this study.

Previous studies on miR‐330 have primarily focused on tumor‐related research. For example, elevated levels of miR‐330‐3p have been reported to promote tumor metastasis in breast cancer by acting on CCBE1, thereby impacting patient survival outcomes (Mesci et al. 2017). Additionally, miR‐330‐3p has been shown to reverse tumor metastasis caused by SPHK1 activation in gastric cancer (Wang et al. 2018). Additionally, miR‐330‐3p plays a significant role in the pathological progression of renal fibrosis by acting on CREBBP (Dai et al. 2025). However, the detailed functions of miR‐330 in OA cartilage and subchondral‐bone tissue have not been systematically reported, and there is limited documentation on the key target genes of miR‐330 in tumors and fibrosis as they relate to OA progression in the previous report. CREBBP was predicted to participate in OA progress by mediating the Notch pathway (Huang, Han, et al. 2025; Huang, Zheng, et al. 2025). While SPHK1 was reported to increase with osteoclast differentiation in OA progression, especially in the subchondral bone (Cherifi et al. 2021), which can upregulate the expression of MMP13 and MMP3. May due to selecting rules' differences, SPHK1 showed no significant differences in our osteoclast RNA sequencing between miR‐330^−/−^ and WT mice. Therefore, we further investigated the mechanism by which miR‐330 regulates cartilage degeneration and bone resorption in OA under the guidance of RNA sequencing based on its histological specificity. Through bioinformatics analysis of the sequencing data, we found that miR‐330 primarily regulates the catabolism hemostasis of chondrocytes and osteoclasts. As the downstream targets regulated by miR‐330, various significant catabolism regulator proteins like FGFR1, CTGF, and EPOR were screened based on the combination analysis of our human joint fluid protein profiling and RNA sequencing changes in miR‐330 global knockout mice. They were further proved to be sensitive to mechanical loading in both previous research and this study (Zou et al. 2024), which was in accordance with the down‐regulation of miR‐330 under mechanical overloading stimulation. Among them, the pivotal role of FGFR1 in OA progression has been extensively documented, as it not only influences cartilage degeneration but also impacts bone destruction (Xie et al. 2020). Targeted small molecule drugs and peptides targeting FGFR1 have shown promising results in OA animal models, as well as in conditions such as osteochondritis and cell death progression (Tan et al. 2018). CTGF is a crucial factor in the regulation of cartilage tissue calcification and hypertrophy in osteoarthritis, impacting the maintenance of cellular matrix homeostasis (Fujisawa et al. 2008; Woods et al. 2009; Tang et al. 2018).

Furthermore, our study has corroborated findings from existing literature by demonstrating through in vitro experiments that the interference of FGFR1 and CTGF can reduce apoptosis in chondrocytes (Fujisawa et al. 2008; Huang et al. 2017; Jiang and Cao 2020). EPOR is divided into two types, as one exists as a membrane receptor, and the other can act as a secreted protein. It has traditionally been believed to bind to EPO and exert a function that promotes red blood cell production. However, recent studies have shown that the expression and activation of EPOR in macrophages can promote cancer metastasis and inhibit macrophage immune function (Chiu et al. 2025), while blocking EPO/EPOR signaling in cancer can effectively eradicate liver tumors in mice (Zhang et al. 2021). Still, reports about the EPO/EPOR axis in osteoarthritis are limited. Few studies have indicated its role in facilitating osteoclast differentiation (Deshet‐Unger et al. 2020; Rauner et al. 2021). The research suggests that the upregulation of EPOR under mechanical stress may contribute to the degeneration of bone tissue in osteoarthritis, which aligns with our findings as accelerating the osteoclast formation. In prior studies, it has been demonstrated that miR‐330 exhibits regulatory effects on the pivotal inflammatory mediator TNF‐α in the progression of OA (Zhu et al. 2020), as well as on the polarization of macrophages (Yang et al. 2024). Through analysis of macrophage RNA sequencing data from miR‐330 global knockout mice and WT mice, it was further observed that miR‐330 also modulates the expression of various inflammatory factors, including IL‐1β and IL‐6 (Yang et al. 2024). The regulatory effects of miR‐330 on the above genes were further verified by the in vivo evidence of conditional knockout mice in chondrocytes and osteoclasts. The absence of miR‐330 in cartilage tissue accelerates both cartilage and subchondral bone degradation. Additionally, suppression of miR‐330 in the macrophage‐osteoclast system led to elevated degeneration of the overlying cartilage tissue. Therefore, the deficiency of miR‐330 can further cause the whole joint inflammation and degeneration.

Our findings clearly indicate that miR‐330 is a potential therapeutic target for OA. In agreement with this, our clinical findings establish that the significant reduction in miR‐330 concentration within synovial fluid demonstrates excellent diagnostic value for predicting OA progression, especially for the early stages without typical MRI bone resorption changes. While intra‐articular delivery of miR‐330 supplements, as a direct therapeutic intervention, effectively mitigated OA progression induced by mechanical instability and protected articular tissues. This coordinated action suppresses the catabolic activation and apoptosis of chondrocytes and inhibits osteoclast activation, thereby blocking the pathological cascade initiated by aberrant mechanical stresses. Consequently, miR‐330 not only provides a sensitive diagnostic window but, in the form of synthetic supplements, emerges as a highly promising biological therapy.

In summary, the most significant finding of this study is the first identification of the mechanoresponsive miR‐330 as a crucial protective factor in OA pathogenesis. We demonstrate that miR‐330 functions simultaneously within both chondrocytes and osteoclasts, regulating their catabolic activity, survival, and inflammatory response under aberrant mechanical stress through the targeted suppression of key mediators including CTGF, FGFR1, EPOR, IL‐1β, and TNF‐α and revealed its underlying downstream mechanism networks with the accordance of TMJ fluid protein profile. However, the upper stream regulation of miR‐330's mechanical sensitivity still remained to be further explored. Critically, the significant downregulation of miR‐330 in synovial fluid, articular cartilage, and subchondral bone of OA patients, coupled with its superior diagnostic performance, establishes it as a robust and novel diagnostic biomarker. Furthermore, our compelling preclinical evidence showed that intra‐articular supplementation of miR‐330 effectively mitigates disease progression in multiple mechanically driven OA models which solidifies miR‐330 itself as a highly promising therapeutic target and agent. Therefore, this work not only unravels a novel mechanobiological pathway underlying OA but also provides a dual‐pronged strategy as both diagnostic biomarker and therapeutic target with significant translational potential for combating osteoarthritis.

## 4 Methods

### 4.1 Synovial Fluid Collection

The collection of all specimens was authorized by the patients and approved by the Ethics Committee of our hospital with informed consent forms signed. Synovial fluid was harvested from TMD patients. Wilkes Stage I (without anterior disc displacement, ADD) was used as a control group. Wilkes stages II‐III (with ADD) and IV‐V (with ADD and OA) were used as experimental groups. ADD can cause abnormal mechanical stress on the condylar cartilage and subchondral bone. The diagnosis of ADD was based on clinical symptoms and MRI.

### 4.2 miRNA and Bulk RNA Sequence

For the rat RNA sequence, total RNA was extracted from 12 rat TMJ OA condyle cartilage and subchondral bone tissues and 6 unmodeled condylar cartilage and subchondral bone tissues by Trizol reagent (Invitrogen, USA) based on the guidance. To meet the requirement of sample qualification, the 2 samples from the unmodeled group and 4 samples from the OA group were randomly combined into one. For the miR‐330^−/−^ mice and WT mice cartilage tissue RNA sequence comparison, the tissues were obtained from the knee cartilage from the 1‐week‐mice of different genotypes. The osteoclasts were obtained from the 6‐week‐old miR‐330^−/−^ mice and WT after M‐CSF and Rankl stimulation. After that, the concentration, purity, and integrity of RNA in each sample were quantified individually. Besides, the RNA library was constructed under the guidance as routine. The sequences of the RNA libraries were offered by Sinotech Genomics Co. Ltd. Biotechnology (Shanghai, China). The data were obtained from an Illumina HiSeq 4000 sequencer, and the quality control obeyed Q30.

### 4.3 Single‐Cell RNA Sequence

For the materials preparation, 8 rats with 16 condyles were included in one sample, including sham with Vector AAV injection, UAC with Vector AAV injection and UAV with miR‐330 AAV injection. The criteria applied to filter low‐quality cells were as follows: gene number < 200 or > 10,000, UMI > 1000, mitochondrial gene proportion > 0.3. Harmony was used to remove batch effects from all samples, a process in which cells are grouped by cell type rather than dataset‐specific conditions (Korsunsky et al. 2019).

To identify the top 2000 variable genes, the filtered gene‐cell matrix was normalized using “LogNormalize” methods in Seurat v.4. PCA was conducted using the top 2000 variable genes, followed by UMAP on the top 30 principal components to visualize the cells. Subsequently, graph‐based clustering was performed on the PCA‐reduced data using Seurat v.4, with a resolution parameter of 0.5 chosen to achieve a more detailed clustering result. Specifically, the first 30 principal components of the integrated gene‐cell matrix were utilized to create a shared nearest‐neighbor graph (SNN) through the FindNeighbors function in Seurat, which was then employed for clustering the dataset via the FindClusters function using a graph‐based modularity‐optimization algorithm based on the Louvain method for community detection.

### 4.4 Elisa

The serum was collected from the fresh blood after setting for 4 h and centrifuging at 4°C for 10 min. The concentrations of serum CTX‐I and PINP were then measured using corresponding ELISA kits, according to the manufacturer's instructions (Mlbio, China).

### 4.5 Primary Chondrocyte Isolation and Micro Mass Assay

Knee cartilage samples were isolated from WT mice and miR‐330^−/−^ mice. Primary chondrocytes were digested with 0.15% collagenase II shaking for 6 h at 37°C. And the following steps was the same as our previous study. F12:DMEM 1:1 (Gibco, USA) supplemented with 10% FBS (Gibco, USA), 100 U/mL penicillin, and 100 μg/mL streptomycin (Gibco, USA) was used as culture conditions in a humidified incubator with 5% CO_2_ at 37°C.

### 4.6 Flow Cytometry

A minimum of 5000 cells per sample were acquired and analyzed via flow cytometry (CytoFLEX, Beckman Coulter) at a rate of 50–500 events per second after staining with Annexin V‐FITC and PI dye for 15 min.

### 4.7 Bone Histomorphometry

At 6 weeks of age, the mice were administered an intraperitoneal injection of 8 mg/kg calcein solution, followed by a 20 mg/kg injection of alizarin red at 7 weeks of age. Subsequently, the mice were euthanized at 8 weeks of age, and their tibias were extracted and embedded in methyl methacrylate (MMA) resin without decalcification. The tibia sections were then sliced to a thickness of 5 μm using a Leica RM2255 microtome and utilized for Von Kossa and TRAP staining procedures. Blank hard slice sections were kept and used for analysis of double fluorescence labeling bone formation. All the trabecular bones under the growth plates of the proximal tibias were analyzed for bone histomorphometric using BIOQUANT OSTEO software (USA).

### 4.8 In Vitro Osteoblast Differentiation Assay

Osteoblast precursor cells were obtained from the calvaria of neonatal mice and cultured in MEM‐α containing 10% FBS (Gibco, USA) and 100 U/mL penicillin and streptomycin (Gibco, USA). For osteogenic differentiation induction, osteoblast precursor cells were seeded in 24‐well plates (8 × 10^4^ cells/well) and stimulated with 10 mM β‐glycerophosphate, 50 μg/mL ascorbic acid and 10^−7^ mM dexamethasone over a 7‐day period. The cells were fixed in 4% PFA for 20 min and stained with alizarin red solution.

### 4.9 In Vitro Osteoclast Differentiation Assay

As previously described, BMMs were isolated from the tibias and femurs of 6‐week‐old mice and were cultured for 5 days in MEM‐α media containing 10% FBS (Gibco, USA), 100 U/mL penicillin and streptomycin (Gibco, USA) as well as 30 ng/mL M‐CSF (Lifetein, China) for cell selection adhesion. For further osteoclast differentiation induction, BMMs were seeded in 48‐well plates (2 × 10^4^ cells/well), then stimulated with 30 ng/mL M‐CSF and 50 ng/mL RANKL (Lifetein, China) for 5 days. Osteoclasts would be fixed in 1% PFA for 20 min and then stained with TRAP solution for 60 min at 37°C.

### 4.10 Quantitative Real‐Time Polymerase Chain Reaction (RT‐qPCR)

TRIzol reagent was used to extract total RNA of chondrocyte, BMMs, and Rat condyle (Invitrogen) according to the offered instruction. All groups of RNA were quantified on a Nanodrop spectrophotometer (Thermo Fisher Scientific, USA). In all, 1 μg qualified RNA with approximate 2.0 value of 260/280 ratio was reverse transcribed into complementary DNA (cDNA) using PrimeScript RT reagent Kit (Yeasen, China) or miRNA First Strand cDNA Synthesis (Tailing Reaction, Sangon Biotech, B532451) under the guidance. The transcription level of related genes was determined using Sybr Green Premix Ex Taq (Yeasen, China) on a Light Cycler 96 instrument (Roche, Basel, Switzerland). Denaturation was set at 95°C for 5 s, followed by annealing at 55°C for 30 s, and extension at 72°C for 30 s, with a total of 40 cycles. Each reaction was performed in triplicate. The mRNA expression level was quantified and normalized to β‐ACTIN and GAPDH by the 2^−ΔΔCt^ method. The micro RNAs expression level was quantified and normalized to U6 or external control by the 2^−ΔΔCt^ method with requirements. The primer sequences were offered by Synbio Technologies (Suzhou/NJ, China) and the primers are listed in Table S2. To compare differences in micro RNAs expression levels during batch screening and validation, the data will be plotted as the ratio of micro RNAs to those of U6 or the external control. When comparing the expression levels of genes of interest following in vitro and in vivo modeling and siRNA transfection, the data will be presented after normalizing the mean values of the control group.

### 4.11 Cell Transfection

Specific target si‐RNA sequences were designed and offered by Genomeditech (Shanghai, China) and transfected respectively into cells with Lipofectamine 3000 (Thermo Fisher Scientific) based on the guidance. The used sequences of targeted si‐RNA are shown in Table S3.

### 4.12 Western Blotting

The cell lysis buffer (Cell Signaling Technology Inc., USA) was used to gain the samples by shaking on ice for 15 min. The samples with loading buffer were subjected to SDS‐PAGE in 10% poly‐acrylamide gels, followed by transferring onto nitrocellulose filter membrane (Millipore, USA). The used primary antibodies were as follows: β‐TUBULIN (1:3000, Proteintech, China), β‐ACTIN (1:3000, Yeasen, China), CTGF (1:1000, Abcam, UK), MMP13 (1:1000, Thermo Fisher Scientific, USA), FGFR1 (1:1000, Cell Signaling Technology Inc., USA), EPOR (1:1000, Thermo Fisher Scientific, USA). After that, the corresponding species‐specific secondary antibodies (HRP‐anti‐rabbit or mouse IgG, Huaxing Bio) were added. β‐TUBULIN or β‐ACTIN was used to normalize the results, adjusting for control variations between individual experiments. The results were detected by the Bio‐rad imaging system (Bio‐rad, USA). The used antibodies and their categories were shown in Table S4.

### 4.13 Interacted Target Gene Prediction and Luciferase Assay

The targeted genes of miR‐330‐3p and miR‐330‐5p were predicted by Target Scan 8.0 and ENCORI both, and further verified by luciferase assay. The targeted genes selection was further supported by the protein mass spectrometry from clinical samples as we previously published (Zou et al. 2024). HEK‐293 T cells, which were purchased from Procell Life Science & Technology Co. Ltd. before, were seeded in 24‐well plates as planned and cultured to 85% confluence till transfection. For the predicted target gene (including ESR1, CTGF, FGFR1, EPOR, IL‐1β) and miR‐330‐3p/5p experiment, 160 ng wild type (WT) and mutant type (MUT) plasmid, and 50 nM of miR330‐3p/5p mimic or negative control were transfected. Every group test was replicated in three parallel wells. After 48 h of incubation, firefly and renilla luciferase activities were detected by a Luciferase Assay Kit (Yeasen, China). Renilla luciferase activities served as an internal reference for results counting, and the value of luminescence of firefly/renilla (termed Luc/Rena) ratios was calculated to show the activity.

### 4.14 Animal Procedures

All the animal study procedure conformed to the ARRIVE Guideline as required. Both rats and mice were anesthetized with Zoletil under the guidance. 60 SD Rats were randomly divided into 2 sham groups with/without Vector AAV injection and 4 unilateral anterior crossbite (UAC) groups, UAC for 8 weeks with injection of Vector AAV and miR‐330 AAV, UAC for 4 or 8 weeks without any other treatment. The rat with UAC for 4 weeks were used in the Rt‐qPCR and FISH experiments to show the miR‐330‐3p/5p expression level in TMJOA in vitro. For the AAV injection models, the rats were kept for 8 weeks. The operation steps of the UAC model were performed as described (Zhu et al. 2020).

All of the mice in this study were on a C57BL/6 background. MiR‐330^−/−^, miR‐330^flox/flox^ as well as the Col2a1‐Cre^ERT2^ mice were purchased from GemPharmatech Laboratories. To generate Col2a1‐Cre^ERT2^; miR‐330^flox/flox^ mice, miR‐330^flox/flox^ mice were mated with Col2a1‐Cre^ERT2^ mice to produce Col2a1‐Cre^ERT^; miR330^flox/−^ mice, which were then mated with miR‐330^flox/flox^ mice. Routine genotyping of tail DNA was performed to confirm cartilage‐specific loss of miR‐330. The similar procedure was applied to the generation of Lyz2‐Cre; miR‐330^flox/flox^ mice. To better illustrate the characteristics of knee OA and TMJOA caused by mechanical instability, the DMM modeling period for the mice was set to be 12 weeks, while the UAC model was set to be 4 weeks. The mice used for all experiments were randomly assigned to control or treatment groups and to those used in OA evaluation.

### 4.15 Micro‐CT Evaluation

The samples of knee and condyle were fixed and underwent assessment using a microcomputed tomography system (SkyScan‐1176, Bruker micro CT, Belgium) with the parameters of 9 μm voxel size. After 3D reconstruction (by NR econ software), the subchondral bone analysis was carried out. The following aspects as BV/TV, Tb.Sp, Tb.Th, and Tb.N were calculated for the spongy and subchondral trabecular bone.

### 4.16 Histological Staining

Rat condyles from each group were fixed with 70% ethanol and prepared for the hard tissue slice and van‐Kossa staining. At least 5 rat condyles as well as mice knee specimens were fixed with 4% paraformaldehyde and embedded in paraffin after EDTA treatment. The paraffin blocks were sectioned at a thickness of 5 μm. Every specimen was first stained with hematoxylin–eosin (HE), 0.1% Safranin‐O (SO) solution, and 0.001% fast green (FG) solution (Sigma‐Aldrich) for primary evaluation and overall view.

### 4.17 Immunohistochemistry (IHC) and Immunofluorescence (IF) Staining

For IHC staining, antigen retrieval was needed in the first step, and it was performed by incubating sections with 0.05% trypsin (pH = 7.8) at 37°C for 20 min. After being blocked with 1% normal goat serum, the sections were then incubated with primary antibodies against FGFR1 (1:200, Cell Signaling Technology, USA), CTGF (1:200, Abcam, UK), COL2A1 (1:200, Millipore, USA), EPOR (1:200, Thermo Fisher Scientific, USA), MMP3 (1:200, Absin, China), MMP13 (1:200, Thermo Fisher Scientific, USA) overnight at 4°C and then for 1 h at 37°C with HRP‐labeled secondary antibodies with biotin for signal amplification (Absin, China). Then DAB was used to stain for 4 min until the brown color appeared in the positive zone. Hematoxylin was used to locate the cell nucleus. For IF staining, after antigen retrieval, it was then blocked and incubated with the primary antibodies for the targeted proteins (FGFR1, 1:200, Cell Signaling Technology, USA; CTGF, 1:200, Abcam, UK; EPOR, 1:200, Thermo Fisher Scientific, USA; IL‐1β, 1:200, Abcam; CTSK, 1:200, Proteintech, China; TNF‐α, 1:200, Abclonal, China) overnight at 4°C. After that, the HRP‐labeled secondary antibodies combined with TSA staining probe or FITC‐labeled secondary antibodies were incubated at room temperature for 1 h. DAPI (Sigma Aldrich) was used to label the cell nucleus.

### 4.18 Fluorescence In Situ Hybridization (FISH)

Cy3‐labeled miR‐330‐3p and FITC‐labeled miR‐330‐5p probes were designed and synthesized by Servicebio (Wuhan, China). The FISH assay of miR‐330‐3p/5p was performed in both human and rat TMJ, mouse TMJ, and mouse knee cartilage and sub‐chondral bone tissues according to the instructions of the manufacturer (Beyotime, China). The cell nucleus was labeled with DAPI (Thermo Fisher Scientific, USA).

### 4.19 Statistics

The statistical analysis was performed utilizing the GraphPad Prism software (version 8, USA). The distribution of data was firstly evaluated by the Shapiro–Wilk test, and the equality of variances was tested using Levene's test. The differences between each two groups in this study were compared by unpaired two‐tailed Student's *t‐*test. To summarize, the data are shown as mean value ± standard deviation (SD) form, and the statistical difference was considered significant when *p* < 0.05*, *p* < 0.01**, *p* < 0.001***; otherwise, ns means non‐significant would be labeled.

### 4.20 Study Approval

Human tissues: All performances were approved by the Human Research Ethics Committee of Shanghai Ninth People's Hospital, Shanghai Jiao Tong University School of Medicine (No. SH9H‐2021‐T141‐1).

All animals used in this study were obtained from Central Laboratory of Shanghai Ninth People's Hospital (Shanghai, China), where rat housing and welfare procedures were performed with the approval from the Institutional Animal Care and Use Committee (IACUC) of Institute of Health Sciences (No. SH9H‐2020‐A56‐1).

## Author Contributions

L.Z. and K.Y. are co‐first authors. L.Z. was involved in the study design, the processed experiments, acquisition of data, and drafting the manuscript. K.Y. helped with the molecular biology, animal experiments and manuscript revision. A.Q. and D.H. contributed to the study protocol revision and revision of the manuscript. C.W. helped with the animal experiments and the figure art work. Y.Y. contributed to the in vitro experiment and revision of the manuscript. X.Z. contributed to the art work and figure design. C.L. and J.Z. helped with sample collection and grouping analysis. All authors declared no conflict of interest, and we all read and approved the final manuscript.

## Funding

This study was supported by grants from the National Natural Science Foundation of China (32071313, 82270996).

## Conflicts of Interest

The authors declare no conflicts of interest.

## Supporting information


**Figure S1:** Both clinical TMJ synovium fluid and Rat condyle tissue micro RNA sequencing showed that miR‐330 was extremely sensitive to mechanical loading.
**Figure S2:** MiR‐330 was down regulated in OA development in clinical specimen and mice UAC models.
**Figure S3:** Conditional knockout of miR‐330 in chondrocytes resulted in the loss of its protective effect on cartilage tissue and cells.
**Figure S4:** Conditional knockout of miR‐330 in osteoclast resulted in bone resorption aggravation and accelerated osteoclast formation.
**Figure S5:** Conditional knockout of miR‐330 in chondrocytes increased the catabolism activation under mechanical stimulation.
**Figure S6:** Conditional knockout of miR‐330 in osteoclast resulted in bone resorption aggravation in vivo *with* instable mechanical loading.
**Figure S7:** Bioinformatic analysis comparison of RNA sequencing of the miR‐330 global knock out mice cartilage and osteoclasts and WT mice.
**Figure S8:** Prediction and screening of miR‐330 targeted genes.
**Figure S9:** IHC staining of miR‐330 targeted genes in conditional knock out mice knee OA model and global knock out mice TMJOA model.
**Figure S10:** Effectiveness of regulating miR‐330 target genes in chondrocytes and osteoclasts.
**Figure S11:** Adeno‐associated virus (AAV) of MiR‐330 for rat TMJ cavity injection and detection.
**Figure S12:** Bioinformatic analysis of sc‐RNA sequencing of miR‐330 AAV treatment effectiveness (gross).
**Figure S13:** Bioinformatic analysis of sc‐RNA sequencing of miR‐330 AAV treatment effectiveness (cell population analysis).
**Figure S14:** GO analysis of sc‐RNA sequencing of miR‐330 AAV targeted genes effectiveness.
**Table S1:** Clinical sample distribution.
**Table S2:** Sequence of primers of RT‐qPCR.
**Table S3:** Targeted SiRNA sequence.
**Table S4:** Antibody category list.

## Data Availability

The datasets used and/or analyzed during the current study are available from the corresponding author on reasonable request. The raw data of sc‐RNA sequencing were uploaded to the GEO database, and the access number is GSE307091.
