# Supplementary material for: Abnormal Stress Reduced miR‐330 Supplementation Alleviates Osteoarthritis Progression by Suppressing Osteochondral Catabolism
Source: Aging Cell. 2026 May 29;25(6):e70553. doi: 10.1111/acel.70553 (PMC13239870; doi:10.1111/acel.70553)
Supplement: Supplementary file 1 — Figure S1: Both clinical TMJ synovium fluid and Rat condyle tissue micro RNA sequencing showed that miR‐330 was extremely sensitive to mechanical loading. Figure S2: MiR‐330 was down regulated in OA development in clinical specimen and mice UAC models. Figure S3: Conditional knockout of miR‐330 in chondrocytes resulted in the loss of its protective effect on cartilage tissue and cells. Figure S4: Conditional knockout of miR‐330 in osteoclast resulted in bone resorption aggravation and accelerated osteoclast formation. Figure S5: Conditional knockout of miR‐330 in chondrocytes increased the catabolism activation under mechanical stimulation. Figure S6: Conditional knockout of miR‐330 in osteoclast resulted in bone resorption aggravation in vivo with instable mechanical loading. Figure S7: Bioinformatic analysis comparison of RNA sequencing of the miR‐330 global knock out mice cartilage and osteoclasts and WT mice. Figure S8: Prediction and screening of miR‐330 targeted genes. Figure S9: IHC staining of miR‐330 targeted genes in conditional knock out mice knee OA model and global knock out mice TMJOA model. Figure S10: Effectiveness of regulating miR‐330 target genes in chondrocytes and osteoclasts. Figure S11: Adeno‐associated virus (AAV) of MiR‐330 for rat TMJ cavity injection and detection. Figure S12: Bioinformatic analysis of sc‐RNA sequencing of miR‐330 AAV treatment effectiveness (gross). Figure S13: Bioinformatic analysis of sc‐RNA sequencing of miR‐330 AAV treatment effectiveness (cell population analysis). Figure S14: GO analysis of sc‐RNA sequencing of miR‐330 AAV targeted genes effectiveness. Table S1: Clinical sample distribution. Table S2: Sequence of primers of RT‐qPCR. Table S3: Targeted SiRNA sequence. Table S4: Antibody category list. [file ACEL-25-e70553-s001.docx]

**Supplementary information**

**Abnormal stress reduced miR-330 supplementation alleviates osteoarthritis progression by suppressing osteochondral catabolism**

Luxiang Zou^1#^, Kaiwen Yang^1#^, Chuyao Wang^1^, Yeke Yu^1^, Xiaoyu Zhang^1^, Chuan Lu^1^, Jieyun Zhao^1^, An Qin^2*^, Dongmei He^1*^

1. Department of Oral Surgery, Ninth People’s Hospital, Shanghai Jiao Tong University School of Medicine; Shanghai Key Laboratory of Stomatology & Shanghai Research Institute of Stomatology; National Clinical Research Center of Stomatology, Shanghai, 200011, China.

2. Shanghai Key Laboratory of Orthopedic Implants, Department of Orthopedics, Ninth People's Hospital, Shanghai Jiao Tong University School of Medicine, Shanghai, 200011, China.

^#^ Luxiang Zou and Kaiwen Yang shared first authorship.

#These authors contributed equally to this work

*Co-corresponding authors

Correspondence to:

An Qin, E-mail: dr_qinan@163.com

Dongmei He, E-mail: lucyhe119@163.com

**Supplementary Figure 1 Both clinical TMJ synovium fluid and Rat condyle tissue micro RNA sequencing showed that miR-330 was extremely sensitive to mechanical loading.**

(A) Typical MRI image of TMJ in different Wilkes stages, the red arrow showed the disc position when mouth closed or open and the green arrow showed the bone resorption in Wilkes IV or V stages.

(B) Raincloud plot showed the over-all changes of miRNAs expression distribution in the joint synovium fluid of clinical samples, showing significant differences between OA and non-ADD group.

(C) Heat map of differentially expressed miRNAs by future sequencing of miRNAs in clinical joint fluid samples.

(D) Heat map of differentially expressed genes by miRNA sequencing in cartilage-cartilage tissue of the rat UAC model.

(E) Volcano map of differentially expressed genes by miRNA sequencing in cartilage-cartilage tissue of the rat UAC model.

(F) Venn plot of clinical joint fluid samples and Rat condylar-cartilage OA and normal control differentially expressed micro RNAs showing that there are 13 micro RNAs were significantly changed in both situations. The micro RNAs not systematically reported in OA development were red labelled.

(G) RT-qPCR comparison of the above 12 selected micro RNAs (except miR-330-3p) under mechanical stimulation in murine chondrocytes (n=4).

(H) RT-qPCR comparison of the above 12 selected micro RNAs (except miR-330-3p) under mechanical stimulation in murine BMMs (n=4).

The error bar represents the mean±SD. ns, nonsignificant. *p<0.05, **p<0.01, ***p<0.001 by unpaired Student’s t test.

**Supplementary Figure 2 MiR-330 was down regulated in OA development in clinical specimen and mice UAC models.**

(A) The ROC curve showed that miR-330-3p meets the diagnosis requirement of ADD related TMJOA development.

(B) The ROC curve showed that miR-330-5p meets the diagnosis requirement of ADD related TMJOA development.

(C) Rt-qPCR results showed the expression of miR-330-3p is significantly higher than miR-330-5p in condyle cartilage and subchondral bone (n=23).

(D) FISH staining of miR-330 in mouse condyle showed that both miR-330-3p (Red) and miR-330-5p (Green) were significantly decreased in UAC leading TMJOA model cartilage (n=5).

(E)The statistical analysis of Figure D, the expression of miR-330-3p/5o is significantly decreased with abnormal mechanical stress in condylar cartilage.

(F) FISH staining of miR-330 in mouse condyle showed that both miR-330-3p (Red) and miR-330-5p (Green) were significantly decreased in UAC leading TMJOA model subchondral bone (n=5).

(G)The statistical analysis of Figure F, the expression of miR-330-3p/5o is significantly decreased with abnormal mechanical stress in condyle subchondral bone.

The error bar represents the mean±SD. ns, nonsignificant. *p<0.05, **p<0.01, ***p<0.001 by unpaired Student’s t test.

**Supplementary Figure 3 Conditional knockout of miR-330 in chondrocytes resulted in the loss of its protective effect on cartilage tissue and cells.**

(A) Comparison of SO staining of knee joints from miR-330^flox/flox^ mice and Col2a1-CKO mice, scale bar=50μm (n=5).
(B) Comparison of SO staining of knee joints from miR-330^flox/flox^ mice and Lyz2-CKO mice, scale bar=50μm (n=5).
(C) IHC staining of COL2A1 and MMP13 in cartilage from miR-330^flox/flox^ mice and Col2a1-CKO mice, scale bar=50μm (n=5).
(D) IHC staining of COL2A1 and MMP13 in cartilage from miR-330^flox/flox^ mice and Col2a1-CKO mice, scale bar=50μm (n=5).
(E) TUNEL staining of comparison of miR-330^-/-^ mice and WT mice knee cartilage showing the apoptosis rate of chondrocytes (n=4).
(F) Quantitative analysis of TUNEL-positive cell rates in Figure E.
(G) TUNEL staining of comparison of miR-330^-/-^ mice and WT mice primary chondrocytes showing the apoptosis rate (n=4).
(H) Quantitative analysis of TUNEL-positive cell rates in Figure G.
(I) Flow cytometry with FITC-Annexin V and PI staining analysis of primary chondrocytes isolated from miR-330^-/-^ and WT mice (n=6).
(J) Statistical analysis of Figure I of cell survival rate.

The error bar represents the mean±SD. ns, nonsignificant. *p<0.05, **p<0.01, ***p<0.001 by unpaired Student’s t test.

**Supplementary Figure 4 Conditional knockout of miR-330 in osteoclast resulted in bone resorption aggravation and accelerated osteoclast formation.**

(A) Micro CT assay of femur spongy bone in miR-330^-/-^ mice and WT mice (n=6 or 7).
(B) The bone volume analysis of miR-330^-/-^ and WT mice spongy bone.
(C) Micro CT assay of femur spongy bone in miR-330^flox/flox^ mice and lyz2-CKO mice (n=3 or 4).
(D) The bone volume analysis of miR-330^flox/flox^ mice and lyz2-CKO mice spongy bone.
(E) Micro CT assay of knee subchondral bone in miR-330^flox/flox^ mice and lyz2-CKO mice (n=3).
(F) Micro CT assay of knee subchondral bone in miR-330^flox/flox^ mice and Col2a1-CKO mice (n=3).
(G) Hard tissue sections combined Von-Kossa staining of miR-330^flox/flox^ mice and lyz2-CKO displaying the subchondral bone volume, scale bar=400μm (n=4).
(H) Hard tissue section with Calcein injection showed new bone formation speed in both miR-330^-/-^ mice and WT mice tibia (n=6).
(I) Analysis of new bone formation rate in tibia of 12 week-age miR-330^-/-^ mice and WT mice.
(J) Alizarin red staining showed calcium nodule formation induced in vitro in osteogenic precursor cells from miR-330^-/-^ mice and WT mice (n=3).
(K) TRAP staining of subchondral bone in knee joints of miR-330^flox/flox^ mice as well as Lyz2 -CKO mice, scale bar=50μm (n=5).
(L) TRAP staining of subchondral bone in knee joints of miR-330^flox/flox^ mice as well as Col2a1 -CKO mice, scale bar=50μm (n=5).
(M) TRAP staining of osteoclast induction of BMMs from miR-330^-/-^ mice and WT mice (n=5).
(N) Statistical analysis of osteoclast formation number and area in Figure M.

The error bar represents the mean±SD. ns, nonsignificant. *p<0.05, **p<0.01, ***p<0.001 by unpaired Student’s t test.

**Supplementary Figure 5 Conditional knockout of miR-330 in chondrocytes increased the catabolism activation under mechanical stimulation.**

(A) Comparison of SO staining of knee joints of miR-330^flox/flox^ mice and Col2a1-CKO mice after DMM operation, scale bar=50μm (n=5).
(B) Comparison of SO staining of knee joints of miR-330^flox/flox^ mice and Lyz2-CKO mice after DMM operation, scale bar=50μm (n=5).
(C) IHC staining of COL2A1 and MMP13 in cartilage of miR-330^flox/flox^ mice with Col2a1-CKO mice after DMM operation, scale bar=50μm (n=5).
(D) IHC staining of COL2A1 and MMP13 in cartilage of miR-330^flox/flox^ mice with Lyz2-CKO mice after DMM operation, scale bar=50μm (n=5).
(E) TUNEL staining of comparison of miR-330^-/-^ mice and WT mice with DMM operation showing the apoptosis rate (n=4).
(F) Statistical analysis of Figure E of positive rate of TUNEL staining that miR-330-/- cartilage with higher sensitivity of mechanical stress loading of cell death.
(G) Flow cytometry with FITC-Annexin V and PI staining analysis of miR-330^-/-^ mice and WT mice primary chondrocytes apoptosis rate with TNF-α treatment (n=4).
(H) Statistical analysis of Figure G of cell survival rate showed that miR-330^-/-^ chondrocytes with higher apoptosis rate of TNF-α stimulation.
(I) Flow cytometry with FITC-Annexin V and PI staining analysis of miR-330^-/-^ mice and WT mice primary chondrocytes apoptosis rate with fluid sheer stress treatment (n=4).
(J) Statistical analysis of Figure I with cell survival rate showed that miR-330-/- chondrocytes with higher sensitivity of mechanical stress loading.

The error bar represents the mean±SD. ns, nonsignificant. *p<0.05, **p<0.01, ***p<0.001 by unpaired Student’s t test.

**Supplementary Figure 6 Conditional knockout of miR-330 in osteoclast resulted in bone resorption aggravation *in vivo with* instable mechanical loading.**

(A) Micro CT assay for characterization of subchondral bone mass in miR-330^flox/flox^ mice and Lyz2-CKO mice after DMM operation (n=5).
(B) Micro CT assay for characterization of subchondral bone mass in miR-330^flox/flox^ mice and Col2a1-CKO mice after DMM operation (n=6).
(C) Hard tissue sections combined Von-Kossa staining of miR-330^flox/flox^ mice and lyz2-CKO micro CT and after DMM-induced knee OA displaying the subchondral bone volume analysis, scale bar=400μm (n=4).
(D) TRAP staining of subchondral bone in knee joints of miR-330^flox/flox^ mice as well as Lyz2 -CKO mice after DMM-induced knee OA, scale bar=50μm (n=5).
(E) TRAP staining of subchondral bone in knee joints of miR-330^flox/flox^ mice as well as Col2a1 -CKO mice after DMM-induced knee OA, scale bar=50μm (n=5).

**Supplementary Figure 7 Bioinformatic analysis comparison of RNA sequencing of the miR-330 global knock out mice cartilage and osteoclasts and WT mice.**

(A-B) Heatmap and volcano diagram displaying RNA-seq differential genes in cartilage tissues of WT mice and miR-330^-/-^ mice.

(C) Venn diagram of the intersection of RNA-seq differential genes in cartilage tissues of WT mice and miR-330^-/-^ mice and downstream target genes of miR-330.

(D) GO analysis of differential genes in the intersected part of Supplementary Figure C, labeled the key genes in the pathway analysis.

(E) KEGG analysis of the intersecting partial differential genes of Supplementary Figure C, labeled the key genes in the pathway analysis.

(F-G) Heatmap and volcano display of WT mouse and miR-330^-/-^ mouse RNA-seq differential genes after osteoclast induction.

(H) Venn diagram of the intersection of WT and miR-330^-/-^ mouse osteoclast RNA-seq differential genes with predicted miR-330 downstream target genes.

(I) GO analysis of intersecting part of differential genes in of Supplementary Figure H, labeled the key genes in the pathway analysis

(J) KEGG analysis of intersecting part of differential genes in Supplementary Figure H, labeled the key genes in the pathway analysis.

**Supplementary Figure 8 Prediction and screening of miR-330 targeted genes.**

(A) Proteomic comparison of joint fluids from clinical OA patients with those from non-ADD patients, box plots showing obvious inter-group differences.

(B-C) Protein heatmaps and volcano plots of differentially expressed proteins in joint fluids from clinical OA patients with those from non-ADD patients.

(D) Target scan and Encori combined predicted downstream target genes that miR-330 may bind in the above differential proteins.

(E) Binding site prediction of the selected targeted gene with miR-330.

(F) Rt-qPCR assay of IL-1β and TNF-α mRNA expression level in BMMs from WT mice and miR-330^-/-^ mice (n=6).

(G) ELISA assay of IL-1β and TNF-α in blood serum from WT mice and miR-330^-/-^ mice (n=6 or 5).

(H) Luciferase assay of binding site of IL-1β and miR-330-5p targeted zone (n=3).

The error bar represents the mean±SD. ns, nonsignificant. *p<0.05, **p<0.01, ***p<0.001 by unpaired Student’s t test.

**Supplementary Figure 9 IHC staining of miR-330 targeted genes in conditional knock out mice knee OA model and global knock out mice TMJOA model.**

(A) IHC staining of CTGF and FGFR1 in miR-330^flox/flox^ mice and Col2a1-CKO mice with or without DMM leading knee OA, scale bar=50μm (n=5).
(B) IHC staining of EPOR, IL-1β and TNF-α with TSA labeling in miR-330^flox/flox^ mice and Lyz2-CKO mice with or without DMM leading knee OA, scale bar=50μm (n=5).
(C) SO and IHC staining of WT and miR-330-/- mice with or without UAC model leading TMJOA of CTGF, FGFR1, CTSK and EPOR, scale bar=50μm (n=5).

**Supplementary Figure 10 Effectiveness of regulating miR-330 target genes in chondrocytes and osteoclasts.**

(A) Post RT-qPCR assay of CTGF, FGFR1, EPOR, IL-1β and TNF-α after targeted knockdown intervention by the targeted si-RNA in chondrocytes and osteoclast respectively (n=5 or 6).

(B) Flow cytometry detection apoptosis of primary chondrocytes gained from WT and miR-330^-/-^ mouse treated by targeted siRNAs (n=5).

(C) Statistical analysis of apoptosis rate Figure H, showed that with interference of miR-330 targeted genes, the apoptosis rate of miR-330^-/-^ chondrocytes could be significantly alleviated.

**Supplementary Figure 11 Adeno-associated virus (AAV) of MiR-330 for rat TMJ cavity injection and detection.**

**(**A-B) Schematic diagram of miR-330 overexpression and Vector AAV construction.

(C-D) FISH staining of miR-330-3p and miR-330-5p showing the comparison of expression in condyle cartilage and subchondral-bone tissues after miR-330 AAV and Vector AAV injection, scale bar= 100μm (n=3).

**Supplementary Figure 12 Bioinformatic analysis of sc-RNA sequencing of miR-330 AAV treatment effectiveness.**

**(**A-C) UMAP display of rat condyle tissue of without model operation with Vector AAV injection, UAC modeling with Vector AAV injection and UAC modeling after miR-330 AAV injection cell clustering.

(D) Chondrocyte, osteoblast, and macrophages-osteoclasts subpopulations and marker gene annotations.

(E) Comparison of chondrocyte, osteoblast, and macrophages-osteoclasts clusters and changes in proportions with UAC modeling and AAV injection treatment.

(F) Violin plots showing the changes in gene expression of different clusters of cells as divided.

(G) Cell heterogeneity analysis in without model operation with Vector AAV injection and UAC modeling with Vector AAV injection groups.

(H) Cell heterogeneity analysis in UAC model operation with Vector AAV injection and miR-330 AAV injection groups.

(I) Heatmap of interested targeting gene expression in different cell clusters.

(J) Typical marker gene expression in chondrocyte population of cells UMAP plot demonstration.

(K) Typical marker gene expression in macrophage-osteoclast population of cells UMAP plot demonstration.

(L) Typical marker gene expression in osteocytes population of cells UMAP plot demonstration.

**Supplementary Figure 13 Bioinformatic analysis of sc-RNA sequencing of miR-330 AAV treatment effectiveness.**

(A)KEGG analysis of differentially up-regulated genes in chondrocyte populations of UAC-modeled rats and unmodeled rats with articular cavity injection of Vector AAV. (B) KEGG analysis of differentially down-regulated genes in chondrocyte populations of UAC-modeled rats with articular cavity injection of miR-330 AAV and Vector AAV injection.

(C) GO analysis of differentially expressed genes regulated by or co-interacted with FGFR1 and CTGF in chondrocyte populations of UAC-modeled rats and unmodeled rats.

(D) GO analysis of differentially expressed genes regulated by or co-interacted with FGFR1 and CTGF in chondrocytes from UAC-modeled rats with articular cavity injection of miR-330 AAV and Vector AAV injection.

(E) KEGG analysis of differentially regulated genes in macrophage-osteoclast populations upregulated in UAC modeled versus unmodeled rats with intra-articular injection of Vector AAV.

(F) KEGG analysis of differential genes down-regulated in the macrophage-osteoclast system of UAC modeled rats injected with miR-330 AAV and Vector AAV.

(G) GO analysis of differentially expressed genes regulated by or co-interacted with EPOR, IL-1βand TNF-α in macrophages-osteoclasts populations of UAC-modeled rats and unmodeled rats.

(H) GO analysis of differentially expressed genes regulated by or co-interacted with EPOR, IL-1βand TNF-α in macrophages-osteoclasts populations from UAC-modeled rats with articular cavity injection of miR-330 AAV and Vector AAV injection.

(I) Heatmap of differential expression gene in osteocytes group of UAC-modeled rats with Vector AAV injection or miR-330 AAV injection and unmodeled rats.

(J) KEGG pathway analysis of differentially up-regulated genes in osteocytes of UAC-modeled rats and unmodeled rats with injection of Vector AAV.

(K) KEGG pathway analysis of differentially down-regulated genes in osteocytes of UAC-modeled rats with Vector AAV injection or miR-330 AAV injection.

**Supplementary Figure 14 GO analysis of sc-RNA sequencing of miR-330 AAV targeted genes effectiveness.**

(A) GO analysis of differentially up-regulated genes in chondrocytes of UAC-modeled rats and unmodeled rats with injection of Vector AAV.

(B) GO analysis of differentially down-regulated genes in chondrocytes of UAC-modeled rats with Vector AAV injection or miR-330 AAV injection.

(C) GO analysis of differentially up-regulated genes in macrophage-osteoclast system of UAC-modeled rats and unmodeled rats with injection of Vector AAV.

(D) GO analysis of differentially down-regulated genes in macrophage-osteoclast system of UAC-modeled rats with Vector AAV injection or miR-330 AAV injection.

**Supplementary Table 1: Clinical sample distribution**

| **Wilkes stages** | **Sample Number*** | **Genders**  **(Female : Male)** | **Age Range (years)** |
| --- | --- | --- | --- |
| I | 28 (6) | 22:6 | 14-71 |
| II | 11 | 9:2 | 16-67 |
| III | 24 | 18:6 | 15-67 |
| IV | 18 (3) | 15:3 | 14-71 |
| V | 15 (3) | 12:3 | 14-68 |

*****The numbers in parentheses indicate the number of samples included in the synovial joint fluid micro sequencing.

**Supplementary Table 2: Sequence of primers of RT-qPCR**

| **Primer name** | **Sequence (5’→3’)** |
| --- | --- |
| Mmu-miR-330-3p | GCAAAGCACAGGGCCTGC |
| Mmu-miR-330-5p | TCTCTGGGCCTGTGTCTTAG |
| Mmu-miR-130a-3p | CAGTGCAATGTTAAAAGGGCAT |
| Mmu-miR-143-5p | TCCTCGGTGCAGTGCTGC |
| Mmu-miR-26b-3p | CCTGTTCTCCATTACTTGGC |
| Mmu-miR-28a-5p | AAGGAGCTCACAGTCTATTGAG |
| Mmu-miR-206-3p | TGGAATGTAAGGAAGTGTGTG |
| Mmu-miR-205-5p | TCCTTCATTCCACCGGAGTC |
| Mmu-miR-196b-5p | TAGGTAGTTTCCTGTTGTTGG |
| Mmu-miR-150-5p | GTGACCATGTTCCCAACCCTCT |
| Mmu-miR-425-3p | ATCGGGAATGTCGTGTCC |
| Mmu-miR-223-5p | CGTGTATTTGACAAGCTGAG |
| Mmu-miR-877-5p | GTAGAGGAGATGGCGCAG |
| Mmu-miR-505-5p | GGGAGCCAGGAAGTATTGATG |
| Hu-miR-330-3p | GCAAAGCACACGGCCTGCAGA |
| Hu-miR-330-5p | CGTCTCTGGGCCTGTGTCTTAGGC |
| Rno-miR-330-3p | GCAAAGCACACGGCCTGCAG |
| Rno-miR-330-5p | CCTCTCTGGGCCTGTGTCTTAGGC |
| Mmu-FGFR1 F | AGAGTCCAAGAGTAAAAGCAGC |
| Mmu-FGFR1 R | CTTCCGAGGTTCAGCTCTCC |
| Mmu-CTGF F | GGCCTCTTCTGCGATTTCG |
| Mmu-CTGF R | GCAGCTTGACCCTTCTCGG |
| Mmu-EPOR F | GGGCTCCGAAGAACTTCTGTG |
| Mmu-EPOR R | TGACTTTCGTGACTCACCCTC |
| Mmu-TNF-α F | CTGAACTTCGGGGTGATCGG |
| Mmu-TNF-α R | GGCTTGTCACTCGAATTTTGAGA |
| Mmu-IL-1β F | GAAATGCCACCTTTTGACAGTG |
| Mmu-IL-1β R | TGGATGCTCTCATCAGGACAG |
| Mmu-GAPDH-F | ACCCTTAAGAGGGATGCTGC |
| Mmu-GAPDH-R | GTTCACACCGACCTTCACCA |
| Mmu-ACTB-F | GCTATGTTGCTCTAGACTTCG |
| Mmu-ACTB-R | GGATTCCATACCCAAGAAGG |

**Supplementary Table 3: Targeted SiRNA sequence**

| **Target** | **Sequence (5’→3’)** |
| --- | --- |
| Mmu CTGF Sense | UUAACUUAGAUAACUGUACAA |
| Mmu CTGF Antisense | GUACAGUUAUCUAAGUUAAUU |
| Mmu FGFR1 Sense | AUAAAACAGCAAAAGUAGCAA |
| Mmu FGFR1 Antisense | GCUACUUUUGCUGUUUUAUUU |
| Mmu EPOR Sense | GUCUGUAUAUAGAAAUAUAUA |
| Mmu EPOR Antisense | UAUAUUUCUAUAUACAGACAG |
| Mmu TNF-α Sense | GUCUCAGCCUCUUCUCAUUCCUGct |
| Mmu TNF-α Antisense | AGCAGGAAUGAGAAGAGGCUGAGACAU |
| Mmu IL-1β Sense | AGCUCUCCACCUCAAUGGAUU |
| Mmu IL-1β Antisense | UCCAUUGAGGUGGAGAGCUUU |

**Supplementary Table 4: Antibody category list**

| **Target** | **Category** | **Application** |
| --- | --- | --- |
| FGFR1 | Cell Signaling Technology, Inc, 9740 | Western Blot, IF, IHC |
| CTGF | Abcam, ab6992 | IF, IHC |
| EPOR | Thermo Fisher Scientific, PA5-91874 | Western Blot, IF, IHC |
| TNF-α | Abclonal, A11534 | IF |
| IL-1β | Abcam, ab283818 | IF |
| CTSK | Proteintech, 11239-1-AP | IHC, IF |
| COL2A1 | Millipore, MAB8887 | IHC |
| MMP13 | Thermo Fisher Scientific, PA5-27242 | IHC |
| MMP3 | Absin, abs130153 | IHC |
| β-ACTIN | Yeasen, 30101ES60 | Western Blot |
| β-TUBULIN | Proteintech, 10094-1-AP | Western Blot |
